# Protocol for extracting flow hydrograph shape metrics for use in time-series flood hydrology analysis

**DOI:** 10.1371/journal.pone.0315796

**Published:** 2025-01-10

**Authors:** Amir Mohammad Arash, Kirstie Fryirs, Timothy J. Ralph

**Affiliations:** School of Natural Sciences, Macquarie University, North Ryde, NSW, Australia; University of Bucharest: Universitatea din Bucuresti, ROMANIA

## Abstract

The shape characteristics of flow hydrographs hold essential information for understanding, monitoring and assessing changes in flow and flood hydrology at reach and catchment scales. However, the analysis of individual hydrographs is time consuming, making the analysis of hundreds or thousands of them unachievable. A method or protocol is needed to ensure that the datasets being generated, and the metrics produced, have been consistently derived and validated. In this lab protocol, we present workflows in Python for extracting flow hydrographs with any available temporal resolution from any Open Access or publicly available gauging station records. The workflow identifies morphologically-defined flow and flood types (i.e. in-channel fresh, high flow and overbank flood) and uses them to classify hydrographs. It then calculates several at-a-station and upstream-to-downstream hydrograph shape metrics including kurtosis, skewness, peak hydrograph stage, peak arrival time, rate-of-rise, peak-to-peak travel time, flood wave celerity, flood peak attenuation, and flood wave attenuation index. Some metrics require GIS-derived data, such as catchment area and upstream-to-downstream channel distance between gauges. The output dataset provides quantified hydrograph shape metrics which can be used to track changes in flow and flood hydrographs over time, or to characterise the flow and flood hydrology of catchments and regions. The workflows are flexible enough to allow for additional hydrograph shape indicators to be added or swapped out, or to use a different hydrograph classification method that suits local conditions. The protocol could be considered a change detection tool to identify where changes in hydrology are occurring and where to target more sophisticated modelling exercises to explain the changes detected. We demonstrate the workflow using 117 Open Access gauging station records that are available for coastal rivers of New South Wales (NSW), Australia.

## 1. Introduction

Streamflow hydrographs integrate spatial and temporal changes in water input, storage and transfer processes within a catchment. Therefore, they contain important quantifiable data that can be statistically analysed and used for a wide range of scientific and applied purposes [1–3]. For example, the shapes of hydrographs can be analysed to reveal the rise and fall, peak stage, travel time and celerity characteristics of flows and floods of different stage heights (water levels) and discharges [2, 4, 5]. When related to channel morphology, such analysis can be used to understand, monitor and assess hydrology during in-channel habitat forming flows or during overbank floods that create floodplains and inundate adjoining land [6].

Streamflow datasets can also be used for catchment and region hydrological classification, temporal and spatial variability analysis of extreme floods, flow peak stages, low flows and baseflows, uncertainty analysis and calibration and validation of hydrologic models, streamflow forecasting and time-series analysis to track changes in flow hydrology as a basis for understanding the impact of climate and anthropogenic disturbances on river systems [2, 7–17]. Across the world there exists tens of thousands of flow gauging stations, some of which contain records extending back decades. The benefits of using real data recorded at gauging stations include a lower level of uncertainty [18], whereas modelled data suffers from limitations associated with the use of a large number of assumptions, capacity to generate long-term data, validation, scalability, accessibility and coding [18–22]. For example, Stahl et al. [23] developed a streamflow records dataset for 441 small catchments in 15 countries across Europe that contained data from 1932 to 2004. This data was used to lab changes in hydrological regimes across these catchments. Parajka et al. [24] analysed 34 studies involving 3,874 catchments to characterise the similarities and differences in hydrology between catchments. This was then used to predict hydrographs in ungauged catchments and analyse underlying climate and landscape controls on their hydrology. Kuentz et al. [25] developed a dataset consisting of 16 flow indicators for 35,215 catchments and 1,366 river gauges across Europe to analyse physical landscape controls on hydrologic variability. Similarly, Brunner et al. [2] used hourly streamflow data from 163 structurally distinct Swiss catchments and 53 years of coverage to characterise flow hydrographs and identify regions of catchments with similar flood reactivity. Arash et al. [26] used over 7,000 hydrographs from rivers in 17 coastal catchments of New South Wales (NSW), Australia to undertake time-series analysis of changes in the hydrological characteristics of in-channel fresh, high flow (bankfull) and overbank hydrology. Gudmundsson et al. [16] investigated global changes in indicators of mean and extreme streamflow from 1951–2010 and 14 subcontinental regions using a data set of observations from over 30,000 sites around the world. Gnann et al. [27] used a MATLAB toolbox (TOSSH) to calculate and validate streamflow time series metrics (e.g. slope of the flow duration curve, mean half flow date, low flow, and runoff ratio etc.) U.S. catchments in that occur within the CAMELS dataset in the USA.

In all of these aforementioned studies, a large number of hydrographs from multiple gauges and spanning long periods of time (years and decades) are needed with which to undertake analyses [5, 28–30]. Traditionally, this was done by direct observation and even manual measurements [31]. However, with the advent of Big Data analysis techniques, scientists can now handle large data sets with wide spatial and temporal coverage and apply analyses in a more consistent and quality-controlled manner. Techniques for hydrograph extraction and analysis such as coding, graphical separating, and digital filtering are now much more widely used [32–35].

The workflow presented in this paper was successfully used in Arash et al. [26] to analyse decadal time‐series changes in flow hydrology to detect whether flood mitigation is occurring. The workflow can be used to extract hydrographs from streamflow time series gauge records and to calculate hydrograph shape metrics for each of them. The workflow has been developed in Python version 3.7. It is user-friendly, simple, quick and informative. Initial steps calculate hydrograph shape metrics at individual gauges, called at-a-station analysis. The metrics calculated include kurtosis (K), skewness (S), peak hydrograph stage (H_p_), and peak arrival time (t_p_) for different flow and flood types. Then, the workflow uses H_p_ and t_p_ to calculate rate-of-rise (RoR) with Excel. Then, ArcGIS Pro version 3.3 [36] is used to calculate contributing catchment area (A_c_), and upstream-to-downstream channel distance between gauges (D). A_c_ and D metrics are then used to calculate upstream-to-downstream metrics including peak-to-peak travel time (t_T_), flood wave celerity (C), flood peak attenuation (H_a_) and flood wave attenuation index (FWAI).

The aims of providing the workflows are to:

1. Improve the utility, and realise the potential of using readily available, Open Access streamflow hydrographs for analysis of changes in flow and flood hydrology at-a-station and from upstream-to-downstream.
2. Provide a method for undertaking time-series analysis of changes in at-a-station and upstream-to-downstream hydrology that can be used to detect where hydrology is changing and where 1D or 2D modelling could be undertaken to explain changes detected.
3. Present a consistent set of workflows that simplifies the process of calculating hydrological characteristics across multiple stations, concurrently and consistently.

## 2. Materials and methods

The protocol described in this peer-reviewed article is published on protocols.io, https://doi.org/10.17504/protocols.io.rm7vzxqo8gx1/v1 and is included for printing as S1 File with this article. A worked example of the hydrograph shape metrics database that is produced is published as a S1 Data on figshare (https://doi.org/10.6084/m9.figshare.27187143).

### 2.1 Study area and gauge records

**Fig 1:**
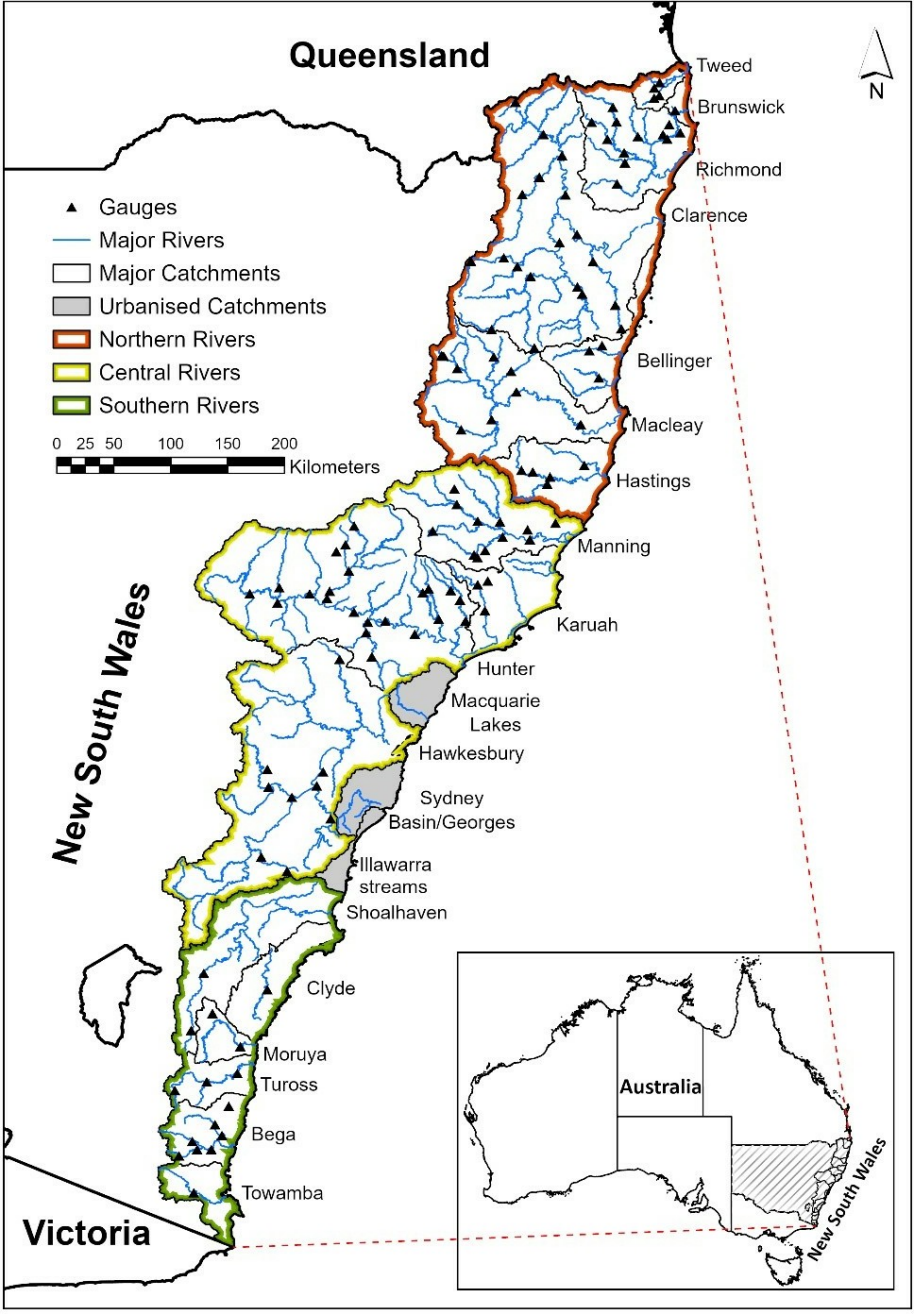
Study catchments and gauge locations used to analyse time series changes in flow and flood hydrology published in Arash et al. [26], and for which the protocol presented in this paper was developed and run. Data source: BoM [42], WaterNSW [43], ESRI [36] and NSW River Styles [44].

Arash et al. [26] presents the full set of results of hydrograph analysis and interpretation of flow and flood changes in 17 out of 20 coastal catchments of NSW, Australia (Fig 1). These coastal catchments receive approximately 1,000 mm to 1,600 mm mean annual rainfall, and the mean daily temperature varies between 18°C to 26°C in summer (December, January and February), and 8°C to 16°C in winter (June, July and August) [37]. Most of these streams flow year-round (perennial), while some streams are more intermittent and others only flow during rainfall events. There are three subregions: Northern Rivers, Central Rivers and Southern Rivers. These subregions experience a variable range of climates due to their vast geographical extent and varying topography [38]. Weather in the subtropical Northern Rivers subregion is wet and mild, and weather in the temperate Southern Rivers subregion is relatively drier and cooler [37]. Since the mid-20th century, there has been a decline of between 8% and 26% in mean annual rainfall and streamflow, especially on the Southern Rivers subregion (with up to 26% decline in mean annual precipitation), and a reduction in the frequency of floods, allowing for riparian vegetation growth in these subregions [30]. However, there will be an increase in intensity and frequency of rainfall and runoff in NSW and across Australia as a result of climate change [39].

Arash et al. [26] focused on understanding time-series changes in flow and flood hydrology for unregulated, rural rivers without large dams or reservoirs, and that have relatively long streamflow records that date back into the early to middle 20^th^ Century. They developed and used the protocol presented here. In that study, 117 gauges were used in catchments that range in size from 513 km^2^ (Brunswick) to 22,333 km^2^ (Clarence). The elevation range of these catchments varies from 27 m to 1538 m, drainage density varies from 0.01 km km^-2^ to 57.07 km km^-2^, and elongation ratio varies from 0.27 (more elongate shape) to 0.53 (more round shape). Of 117 gauges, 27 gauges are located in confined rivers, 72 in partly confined rivers and 17 in laterally-unconfined rivers. Generally, rivers in confined settings are located in the upstream gorges, rivers in partly-confined setting are located in the upstream gorges, and rivers in laterally-unconfined setting are located in low-lying coastal areas [40, 41]. The analysis used Open Access streamflow data sourced from the Australian Bureau of Meteorology (BoM) [42], and WaterNSW [43]. The streamflow data in these databases is provided in various time intervals, from 15 minutes, to hourly, and daily.

### 2.2 Hydrograph analysis and quantification

**Fig 2:**
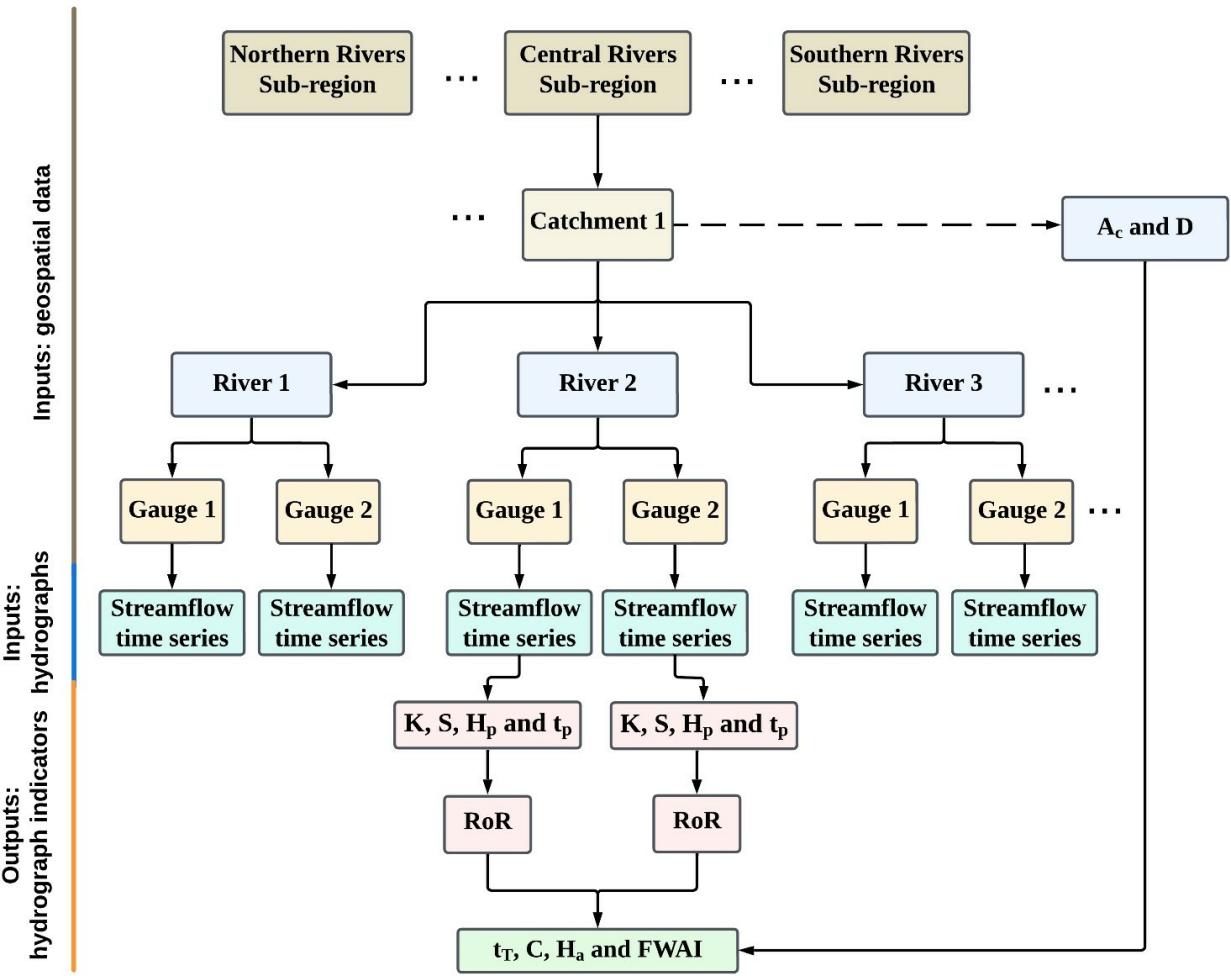
Hierarchical structure of the hydrograph analysis protocol. Data source: BoM [42], WaterNSW [43], ESRI [36] and NSW River Styles [44].

Fig 2 shows the hierarchical structure of the hydrograph analysis protocol presented in this study. In this protocol, we quantify hydrograph shape metrics directly from streamflow time series data at individual gauges (at-a-station) and from upstream-to-downstream (Fig 2). Flow stage hydrographs are the key inputs required to run the protocol (Fig 2). The at-a-station output metrics are kurtosis–K (Eq 1), skewness–S (Eq 2), peak hydrograph stage–H_p_, and peak arrival time–t_p_. Hp and t_p_ are then used to calculate rate-of-rise–RoR (Eq 3) (Fig 2). The upstream-to-downstream analysis focuses on the moment of peak flow production. For upstream-to-downstream analysis, two gauges on the same streamline are required. The output metrics of peak-to-peak travel time (t_T_) (Eq 4), flood wave celerity (C) (Eq 5), flood peak attenuation (Ha) (Eq 6) and flood wave attenuation index (FWAI) (Eq 7) are calculated within Excel (Fig 2). For the upstream-to-downstream analysis, additional geospatial data is needed as an input (Fig 2), including contributing catchment area (A_c_) at the gauge, and channel distance between the gauges (D).


$$Kurtosis=\frac{\mu_{4}}{\sigma^{4}}$$ (Eq 1)

$$Skewness=\frac{\mu_{3}}{\sigma^{3}}$$ (Eq 2)

$$RoR=\frac{H_{p}}{t_{p}}$$ (Eq 3)

$$t_{T}={\left[t_{p}\right]}_{Upstream}-{\left[t_{p}\right]}_{Downstream}$$ (Eq 4)

$$C=\frac{D}{t_{T}}$$ (Eq 5)

$$H_{a}={\left[H_{p}\right]}_{Upstream}-{\left[H_{p}\right]}_{Downstream}$$ (Eq 6)

$$FWAI=\frac{{\left[H_{p}\right]}_{Upstream}}{{\left[A_{c}\right]}_{Upstream}}-\frac{{\left[H_{p}\right]}_{Downstream}}{{\left[A_{c}\right]}_{Downstream}}$$ (Eq 7)

Where μ refers to the arithmetic mean; μ_3_ corresponds to the third moment about the mean; μ_4_ corresponds to the fourth moment about the mean; Ϭ refers to the standard deviation; RoR refers to rate of rise (m hr^-1^); H_p_ refers to the peak hydrograph stage (m); t_T_ refers to peak-to-peak travel time (hr); t_p_ refers to the peak arrival time (hr); H_a_ refers to flood peak attenuation; FWAI refers to flood wave attenuation index; A_c_ refers to the contributing drainage area (km^2^); C refers to the peak-to-peak flood wave celerity (m s^-1^); and D refers to the upstream-to-downstream channel distance between gauges (km).

At-a-station analysis can be conducted on any single station data. However, for upstream-to-downstream analysis, two gauging stations need to be located along the same streamline. To synchronise the flow and flood dynamics along the channel or valley over time, we recommend that the peak arrival time (t_p_) values be based on the physical distance between the upstream and downstream gauges along the channel, and the calculated C values. Given that C values commonly range between 0.25 m s^−1^ and 10 m s^−1^ [45–47], any values outside this range can be considered outliers. This indicates that the flood event at the upstream gauge is independent of the recorded flood event at the downstream gauge, making them unsuitable for pairing in upstream-to-downstream analysis. However, these flood hydrographs can be separately used for at-a-station analysis.

A_c_ was calculated using a 30-m SRTM DEM using the flow direction and flow accumulation tools (ArcHydro Extension) within ArcGIS Pro. These tools indicate the flow direction in each streamline and accumulate all flows from upstream into gauges (pour points). The watershed tool was used to delineate subcatchments draining into each gauge, and the calculate geometry tool was used to calculate A_c_ for each gauge. D was calculated using the major and minor streamline and gauge location shapefiles in the NSW River Styles dataset [44], and calculate geometry tool in ArcGIS Pro, but any polyline stream network database can be used. In our study catchments, A_c_ varies from 26 km^2^ to 17,320 km^2^, and D ranges from 14 km to 118 km [26], demonstrating the range of catchment sizes that the workflow can be used on. FWAI is a dimensionless indicator of flood peak attenuation that is normalised by A_c_ at the upstream and downstream gauges. This indicator accounts for the lateral inflow from tributaries (a gaining reach) with negative FWAI and lateral outflow from tributaries (a losing reach) with positive FWAI [48].

### 2.3 Determine flow stage classes

Hydrologists and river experts often classify flow stage heights into categories based on water level at cross-sections. This protocol runs on stage height hydrographs instead of discharge for several reasons including, greater data availability, ease of comparison across different streams, ease of visualisation, and to match to stakeholders’ experience in the field and emergency warnings that are issued during floods [49]. Also, each river has a different morphology and therefore discharge or recurrence intervals for a bankfull flow are not consistent across the landscape and river morphology can change over time. McMahon and Peel [50] analysed 622 rating curves from 171 gauges and found that uncertainties in BoM dataset’s stage–discharge for daily streamflow ranges from −4.2% to 4.5%, therefore we use stage height hydrographs instead of discharge hydrographs.

**Fig 3:**
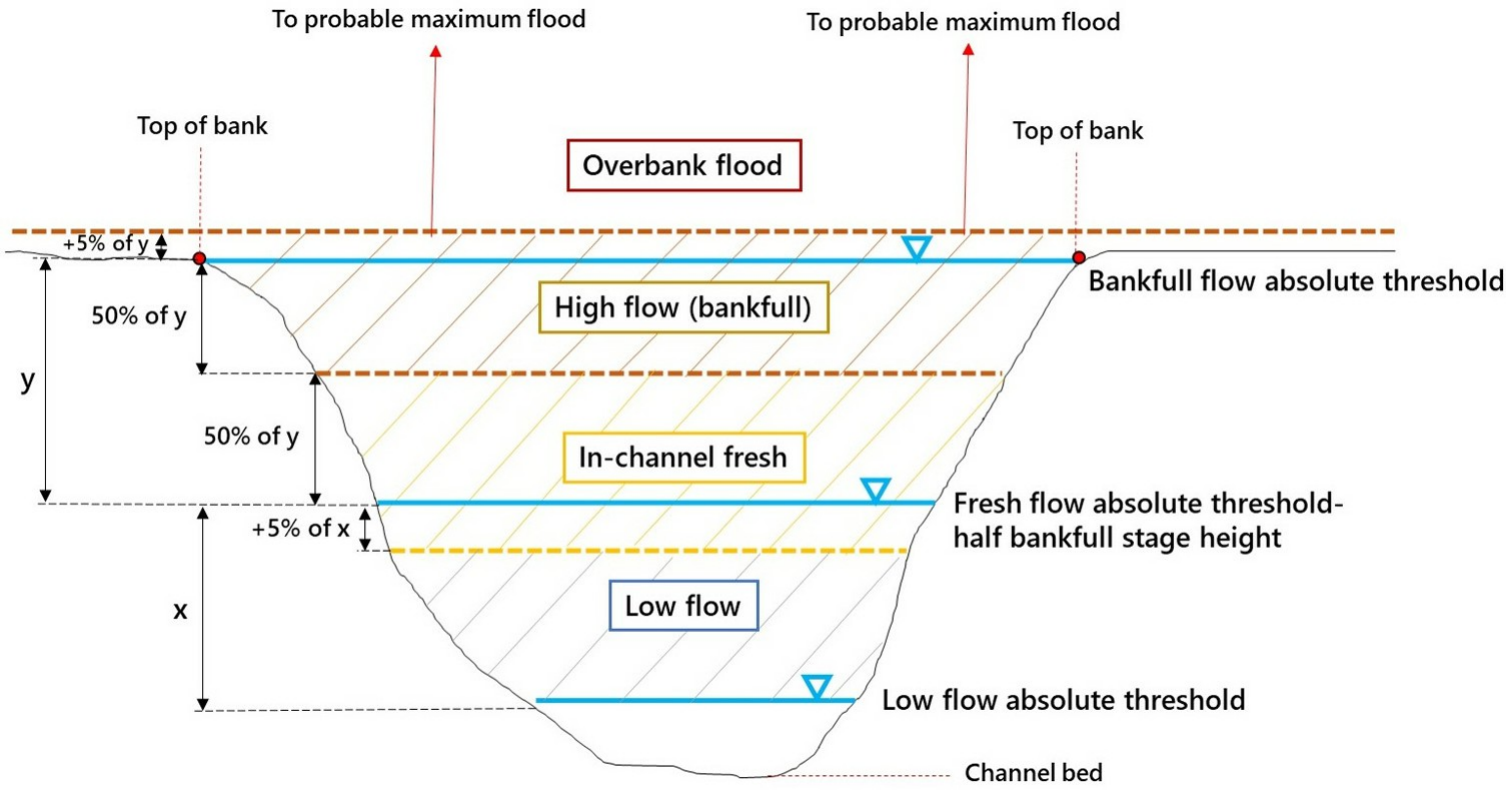
Schematic cross-section showing stage height thresholds and flood stage classes that can be used in the protocol (sourced by Arash et al. [26]).

This protocol runs on three morphologically-defined flow and flood stages which can then be used to undertake like-with-like comparisons of flow and flood behaviour across catchments and regions. The three flow stages are: in-channel fresh, high flow and overbank flood (Table 1 and Fig 3).

**Table 1:** Definitions of different flow stages [26, 42, 51].

Flow stage class	Definition of the flow stage
**In-channel fresh**	A stage height that is 95% higher than the low flow absolute threshold and 50% higher than the fresh flow absolute threshold (half bankfull stage height).
**High flow**	A stage height that is 50% lower than the bankfull flow absolute threshold (top of the macrochannel bank) and 5% higher than bankfull flow absolute threshold.
**Overbank flood**	A stage height that is 5% above the bankfull flow absolute threshold.

In-channel freshes are defined as flows that are larger than low flows (or baseflows). They may express as a noticeable rise in streamflow height that lasts for a few days, triggered by short bursts of rain [51]. These flows interact with submerged hydraulic units and low-elevation instream geomorphic units.

A high flow is defined as the bankfull water level (the top of the macrochannel bank). These flows interact with submerged hydraulic units and low-elevation instream geomorphic units.

Overbank floods are flows that completely fill the macrochannel and overflow onto floodplains. Any flow higher than bankfull flow level is considered an overbank flood. These flows connect channels to floodplains and interact with floodplain features, including riparian vegetation and wetlands.

To identify these flow and flood types using the workflow, morphological thresholds are first identified at each gauge station site. These thresholds and flow stage heights are manually calculated in Excel and use Python code. Table 1 contains definitions of these different flow stages and Fig 3 shows a schematic cross-section with stage height thresholds and flood flow types indicated.

The low flow absolute threshold is calculated in excel by using the flow frequency analysis and rating curves provided with gauge data (in our case from BoM [42] and WaterNSW [43]), and the US EPA 7Q10 approach [52]. 7Q10 is defined as the lowest 7-day average flow that is expected to occur once every 10 years, on average [52]. Here, low flows (or baseflows) are defined as sustained low-level flows that exceed low flow absolute threshold. The in-channel fresh absolute threshold is defined as half bankfull stage height. High flows are defined as flows that exceed the in-channel fresh level, but are restricted to bankfull level. Overbank floods are any flow that exceeds the bankfull flow absolute threshold by 5% or more. For example, based on BoM [42], Hunter River at Singleton (gauge ID: 210001) experienced an overbank flood in 09/03/2022 as a result of a 4-day precipitation event with accumulated rainfall of 125 mm (recorded at Glennies Creek rainfall gauging station), a high flow in 10/12/2021 as a result of a 3-day precipitation event with accumulated rainfall of 70 mm, and an in-channel fresh in 13/11/2021 as a result of a 2-day precipitation event with accumulated rainfall of 58 mm.

### 2.4 Extraction and quantification of flow hydrograph metrics from streamflow time series

**Fig 4:**
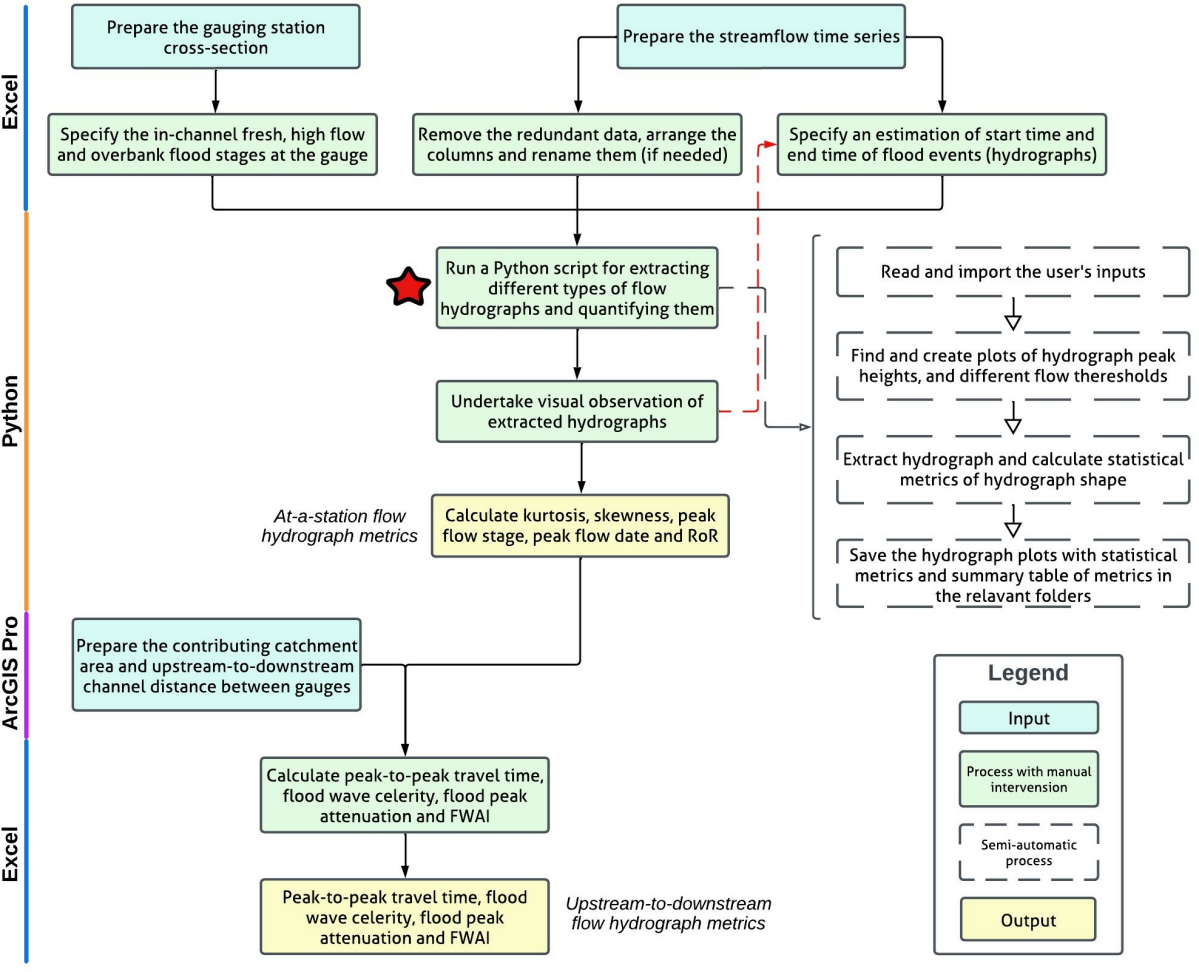
Flowchart showing the full methodology for extracting and quantifying hydrographs from the streamflow time series at each gauge. The yellow boxes produce a metrics database as an output. The Python script indicated by a red star, is detailed in the protocols.io that accompanies this paper (https://doi.org/10.17504/protocols.io.rm7vzxqo8gx1/v1). Data source: BoM [42], WaterNSW [43], ESRI [36] and NSW River Styles [44]. Green boxes and white boxes denote parts of the protocol that require manual intervention by a user and semi-automatic parts, respectively.

The workflow to quantify flow hydrograph metrics both at-a-station and from upstream-to-downstream is shown in Fig 4. This workflow contains 10 main steps:

1. Prepare input data including the hydrographs and geospatial data in Excel (Fig 2).
2. Process gauging station cross-sections to identify the in-channel fresh, high flow and overbank flood stages at each of the gauges in Excel.
3. Input the streamflow time series hydrographs from gauge records into Excel (in NSW these are sourced from the BoM [42] and WaterNSW [43]).
4. Remove redundant data in the excel file of streamflow timeseries.
5. Specify the start and end time of the flow or flood event using the hydrographs in Excel.
6. Run the Python script to extract in-channel fresh, high flow and overbank flood hydrographs.
7. Verify the hydrograph extraction process by visual observation and adjust the inputs (remove outliers and adjust start time and end time) if needed in the Python script.
8. Calculate hydrograph metrics; kurtosis (Eq 1), skewness (Eq 2), peak flow stage, peak flow date and rate-of-rise (RoR) (Eq 3) for each hydrograph in Python.
9. Calculate contributing catchment area (A_c_), and upstream-to-downstream channel distance between gauges (D) in ArcGIS Pro and input them into the Python script (in NSW these metrics are calculated by using NSW River Styles streamlines layer [44] in ArcGIS Pro [36]).
10. Calculate peak-to-peak travel time (t_T_) (Eq 4), flood wave celerity (C) (Eq 5), flood peak attenuation (H_a_) (Eq 6) and flood wave attenuation index (FWAI) (Eq 7) in Excel.

Please note that Steps 8 and 10 produce a hydrograph metrics database as an output (See Fig 2).

In different places and for different gauges, stage height data may come to a user in different formats. Therefore, setting up the input data excel spreadsheet to a standard format is necessary. For example, the streamflow data download from BoM [42] and WaterNSW [43], portals for NSW streams needs to be adjusted and filtered before importing into the Python code. The label of the first column and row (cell A1) should be ‘date’ and the second column first row (B1) should be ‘value’. All other redundant information should be removed from the excel file. Then, this file should be saved in Microsoft Excel .xlsx format. Khaleghi et al. [53] showed 1-hr geomorphoclimatic unit hydrograph can be used in accurately simulating flow hydrograph and quantifying them using shape metrics (e.g. peak discharge and time-to-peak). Hence, the workflows use the 1-hour interval date data. The format of the date is “dd/mm/yyyy hh:mm:ss”, and the unit of stage height is meters. There is a possibility of using data with coarser (2-hour, 3-hour etc.) or finer date (30-minute, 15-minute etc.) intervals. The users can directly change the time interval in the Python code (See Protocols.io), or they can change their time-series to downscale or aggregate to hourly by using appropriate statistical methods, such as StreamFARM algorithm [54].

Preparation of other inputs include identification of the in-channel fresh, high flow and overbank flood stages at each gauge station and identification of the start time and end time of each flow or flood event of interest. The start time would typically be the time at which a precipitation event causes a rise in stage height. The end time normally indicates a return to base flow level (low flow level). To identify the start and end times, flow record variability, local flow conditions and the purpose of study should be considered. These are user decisions that need to take place at this stage of the protocol. Recognising that the duration of flows may vary in other landscapes, the start and end dates can be changed in the protocol. The only criteria are to ensure the full presentation of hydrograph is preserved in the output. For example, local-knowledge about flood dynamics can be used to define the duration of floods in the climatic and environmental settings where the protocol is being applied. It could be possible to use the time between the start of rainfall and onset of flooding as a reference for this purpose. Other users may wish to trial different durations by running the protocol multiple times to test any effect on the output. In our eastern NSW study, the identification of start and end dates for extracting hydrographs is undertaken using visual observation of the extracted hydrographs and knowledge of how floods of different magnitude function in this landscape. In the Arash et al. [26] study, 10 days was selected as the duration of flow and flood events. For ease, this is defined as 5 days back from the flow peak and 5 days forward from the flood peak (See protocols.io).

By running the Python code, the script extracts flow hydrographs and calculates kurtosis, skewness, peak value, peak date and some other statistics for each hydrograph. Before undertaking further analysis, the extracted hydrographs need to be checked to ensure that the delineated hydrographs and calculated metrics are correct and any inputs (streamflow time series, flow stages, and duration of hydrographs) adjusted where needed.

In Microsoft Excel, RoR is calculated using peak value and peak date in Eq 3. Total drainage area draining into each gauge and total area of each catchment is calculated using the watershed tool in ArcHyro extension in ArcGIS Pro [26]. Subcatchment area draining to each gauge is classified as small (e.g. < 2,000 km^2^), moderate (e.g. 2,000–10,000 km^2^) and large (e.g. > 10,000 km^2^). Also, gauges are classified as upstream (a gauge captured 10% of the total catchment area), midstream (11%– 50%), and downstream (> 51%).

Peak-to-peak travel time of each pair of hydrographs is calculated on the peak value at the upstream and downstream gauges. The distance between upstream and downstream gauge pairs is calculated in ArcGIS Pro by using any readily available, accurate, streamlines layer (in NSW this is the River Styles streamlines layer [44]). The distance between gauge pairs and peak-to-peak travel time are used to calculate flood wave celerity. FWAI is calculated using the peak stage height value and contributing drainage area at the upstream and downstream gauges.

The flow hydrographs, flow stage classes and Microsoft Excel file with descriptive statistics for each flow hydrograph are stored in corresponding folders (‘Overbank_flood_peaks’, ‘High_flow_peaks’, ‘In_channel_fresh_peaks’). Moreover, the computed summary statistics (count, mean, std, min, 25%, 50%, 75%, max, kurtosis, skewness, start_date, peak_date, end_date) for each flow stage class are saved as CSV files (In_Channel_fresh_peaks_sats.csv, High_flow_peaks_sats.csv and Overbank_flood_peaks_sats.csv). This enables users to undertake subsequent analysis, visualisation, and storage of data and hydrographs derived from the streamflow data, by organising it into separate folders and files.

## 3. Expected results and technical validation

**Fig 5:**
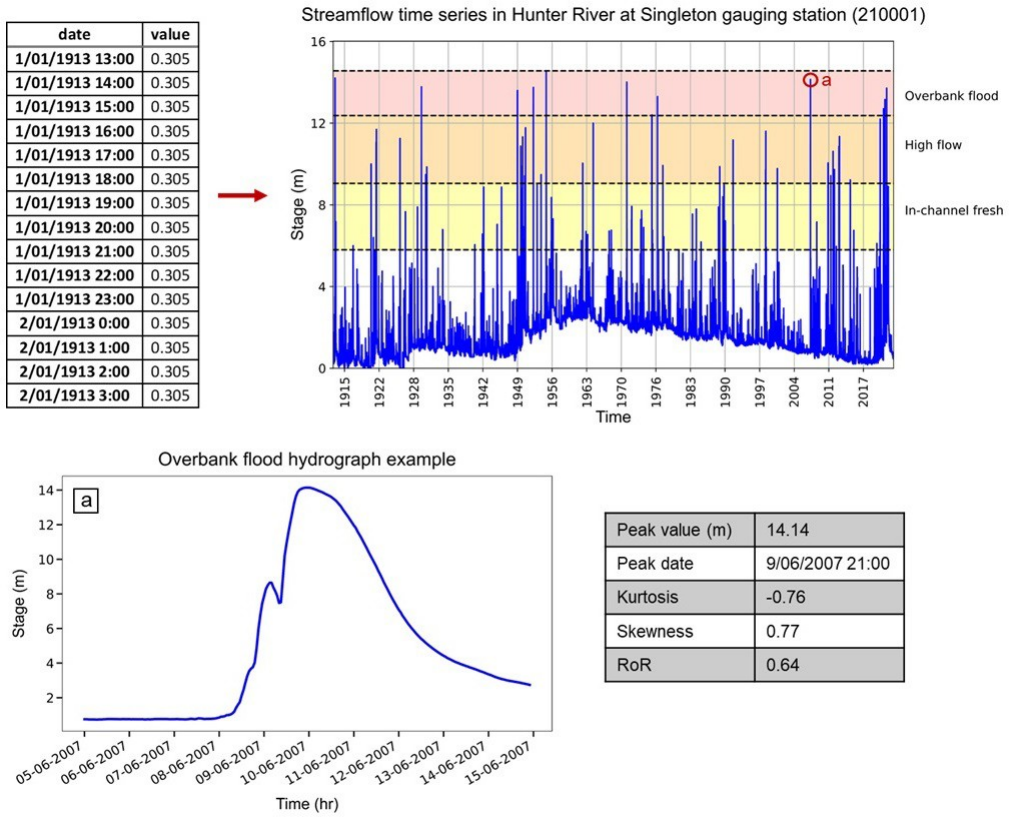
An example of script results used to extract flow hydrographs from streamflow time series for the Hunter River at Singleton gauging station. The red circle labelled “a” is the 2007 flood. Data source: BoM [42], WaterNSW [43], ESRI [36] and NSW River Styles [44].

In the Arash et al. [26] study, the hourly interval streamflow time series of 117 gauges across coastal catchments of NSW were used to extract hydrographs for different flow stage classes (Fig 1). The total number of in-channel fresh, high flow and overbank flood hydrographs is 4,600, 1,584 and 868, respectively. Overall, 3,371 hydrographs occur in the Northern Rivers region, 3,008 in the Central Rivers region and 673 in the Southern Rivers region (Fig 1). Fig 5 shows an example of the application of script to extract and quantify flow hydrographs for the in-channel fresh, high flow and overbank flood flow stage classes from streamflow time series data for the Hunter River at Singleton gauging station from 01/01/1913 to 21/11/2023.

The script is executed iteratively, allowing for manual verification of the generated hydrographs. This ensures that the specified inputs consistently produce hydrographs that align with real-world expectations. Additionally, thorough checks are implemented to prevent the duplication of hydrographs, and mixed results in the output dataset. For our NSW study areas, the streamflow time series sourced from BoM [42] and WaterNSW [43] are of high quality and are run through various QA/QC checks within these agencies before release [50]. For example, if a gauge has been moved there is a calibration process put in place to ensure that the gauge sensors are recording the same stage height throughout the record. In other places, a user needs to undertake their own verification and QA/QC checks on the input data they are using before running the protocol. The protocol itself does not do QA/QC or uncertainty analysis for a user. Such analyses should be conducted ex-post or ex-ante the protocol being run.

## 4. Applications and limitations of the protocol

Hydrographs are valuable sources of information for understanding the spatial and temporal patterns of water flowing through the landscape and how this varies over time and in response to specific events at different locations in catchments. The data that underpins hydrographs can be used to calculate a range of meaningful hydrological metrics, and to undertake statistical analyses, to further understand the hydrological function of rivers and their catchments. Like elsewhere in the world, Australia has online data portals such as the Bureau of Meteorology (BoM) [42] and WaterNSW [43], that hold Open Access streamflow gauging station records, including extensive records of flood hydrographs that in some cases have a time series dating back many decades. The extraction and quantification of hydrographs using these gauging records can be used to better understand hydrological processes for a range of scientific and applied purposes [1, 2, 26, 31].

Applications of the semi-automated hydrograph data extraction and quantification methods presented in this lab protocol (and see protocols.io) and the workflows within it, can be broadly grouped based on their purpose: current understanding (i.e. present, contemporary focus), hindcasting (i.e. historical, past focus), or forecasting (i.e. predictive, future focus). For studies with a contemporary focus, users can run the protocol and workflows provided here to extract hydrographs from current and recent historical time-series gauge records and to calculate at-a-station and upstream-to-downstream hydrograph shape metrics. At present, these metrics include kurtosis, skewness, peak hydrograph stage, peak arrival time, rate-of-rise, peak-to-peak travel time, flood wave celerity, flood peak attenuation, and flood wave attenuation index. The method is adaptable and has potential for customisation by incorporating additional flow hydrograph metrics, thereby allowing for further improvements to suit user preferences and research objectives. It facilitates the uptake and use of existing flow hydrograph databases (both real-time flow data and historical flood records), while maintaining flexibility to add new flow time-series records as they become available.

The protocol uses statistical and machine learning methods to identify patterns, trends, and anomalies in data [55–57]. The outputs produced by the protocol can also be integrated and used in various GIS and hydrological model applications, to help build a more comprehensive understanding of hydrological processes in catchments [58–61]. The protocol may be employed to produce user-friendly summary data or databases without overcomplicated analysis [62–64]. Users can conduct quality checks on the data to make sure that it accurately reflects the current conditions [65–67] and matches on-ground observations and experiences of community, particularly during floods [68–70].

Use of the protocol enables implementation of standardised formats and methods for collecting, analysing, and sharing hydrograph data [71–73]. Using standardised methods to assess hydrographs across rivers, catchments, regions, and even internationally, allows robust comparison of key metrics that has previously been restricted to coarse-data or modelling approaches. The protocol provides hydrograph analysis tools and makes them accessible to authorities, researchers, and the public [74, 75]. Analysis of outputs derived from use of the protocol can facilitate research in hydrology and river management or be applied by managers in flood monitoring and management [65, 67, 76]. Simple hydrograph datasets can also be used to prepare visualised educational materials to raise public awareness about flood risks and flood hydrology [77–79].

The outputs produced using the protocol can be used to make better interpretations of past and emerging patterns and changes in the flow hydrology at-a-station, from upstream-to-downstream on key streamlines, or across different catchments and regions. In this regard, the protocol could be considered a change detection tool to identify where changes in hydrology are occurring and where to target more sophisticated modelling exercises to explain the changes detected. Hence, it facilitates hindcasting and forecasting of hydrological processes using an efficient and cost-effective method for generating streamflow metrics. Hindcasting efforts may use extracted hydrograph metrics from modern rivers to help reconstruct conditions in catchments prior to current hydrological gauge records. This may be achieved by identifying catchments with similar environmental setting and fluvial controls on hydrology, and then extrapolating the most likely hydrograph characteristics in the past for similar catchments. In terms of forecasting, the protocol may be used to help predict future hydrograph size and shape in catchments. This understanding can help aid river and flood modelling, planning and management, and for the detection of hydrological impacts of any river recovery or natural flood management rehabilitation activity [76–83]. Overall, the protocol can be universally applied to any streamflow gauging records, providing standardised processes to compare flow hydrograph characteristics at site, reach, catchment, and regional scales, allowing for many applications in natural sciences.

There are some considerations that users need to be aware of when using the workflow and protocol.

First, in the data preparation step, 1-hour interval stage height is used. To run data with different intervals, users can change the Python code, or downscale or aggregate the time-series from the default date interval to sub-daily or hourly by using other statistical methods (e.g. [54, 84]).

Second, given the diverse hydrological, meteorological, and geomorphic characteristics of rivers around the world, choosing the start and end dates for the extraction of hydrographs is a decision that needs to be based on visual observation of the extracted hydrographs and local knowledge, with the proviso that the full hydrograph is preserved in the output. Other approaches to identifying start and end dates have been provided above.

Third, the hydrograph shape metrics calculated by the protocol capture multiple flow characteristics (such as flood peak height, time-to-peak, the steepness of the rising and recession limbs, and flatness of the peak). However, the workflow can be tailored to include other hydrograph shape metrics that may be of interest to a user. This can be done manually in Excel at the data preparation stage. For upstream-to-downstream hydrograph shape metrics (e.g. C and t_T_) this is done by using the provided Python code, and for catchment morphometrics (e.g. A_c_ and D) in ArcGIS Pro.

Finally, while the incorporation of rainfall data into hydrograph characterisation analysis could aid understanding of the (de)synchroneity of flood wave propagation from upstream to downstream, the protocol does not quantify nor consider the timing of precipitation or the magnitude of precipitation events. Analysis of lag time between rainfall, runoff and the expression of flow in river channels, preconditioning of the landscape and the role of disparate contributions from tributaries or episodes of rainfall in different parts of catchments are not considered within the scope of the protocol presented here. Further work and expansion of the protocol would be needed to detect, quantify or account for these factors.

## 5. Conclusions

In this protocol, and associated protocols.io (https://doi.org/10.17504/protocols.io.rm7vzxqo8gx1/v1), we provide a set of workflows to extract hydrographs from time-series gauge records that can be used to calculate at-a-station and upstream-to-downstream hydrograph shape metrics. The protocol employs efficient coding, supported by relevant analytical libraries, and includes comprehensive, easy-to-follow comments. The applications of this protocol include as a change detection tool to identify where changes in hydrology are occurring and where to target more sophisticated modelling exercises to explain the changes detected, to visualise and analyse flow hydrograph data to enhance the understanding of data types, structures, missing values, and potential biases while uncovering relationships, trends, and patterns over space and time. This method is designed to be user-friendly, thereby facilitating wider application in various hydrological projects.

## Supporting information

S1 Data This spreadsheet is available on figshare (https://doi.org/10.6084/m9.figshare.27187143). (XLSX)

S1 File Step by step protocol. (PDF)
