# Supplementary material for: Protocol for extracting flow hydrograph shape metrics for use in time-series flood hydrology analysis
Source: PLoS One. 2025 Jan 10;20(1):e0315796. doi: 10.1371/journal.pone.0315796 (PMC11723534; doi:10.1371/journal.pone.0315796)
Supplement: S1 File — (PDF) [file pone.0315796.s002.pdf]

Dec 05, 2024

Protocol for extracting flow hydrograph shape metrics for use in time-series flood hydrology analysis

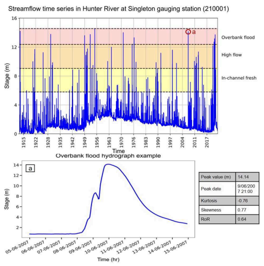

DOI

dx.doi.org/10.17504/protocols.io.rm7vzxqo8gx1/v1

Amir Mohammad Arash[1], Kirstie Fryirs[1], Tim J. Ralph[1]

[1]Macquarie University

Amir Mohammad Arash

Macquarie University

OPEN ACCESS

DOI: **dx.doi.org/10.17504/protocols.io.rm7vzxqo8gx1/v1**

Protocol Citation: Amir Mohammad Arash, Kirstie Fryirs, Tim J. Ralph 2024. Protocol for extracting flow hydrograph shape metrics for use in time-series flood hydrology analysis. **protocols.io** **https://dx.doi.org/10.17504/protocols.io.rm7vzxqo8gx1/v1**

Manuscript citation:
Arash, A. M., Fryirs, K., & Ralph, T. J. (2023). Detection of decadal time-series changes in flow hydrology in eastern Australia: Considerations for river recovery and flood management. *Earth Surface Processes and Landforms*. https://doi.org/10.1002/esp.5694

Fryirs, K., Zhang, N., Ralph, T. J., & Arash, A. M. (2023). Natural flood management: Lessons and opportunities from the catastrophic 2021–2022 floods in eastern Australia. *Earth Surface Processes and Landforms*, *48*(9), 1649-1664. https://doi.org/10.1002/esp.5647

License: This is an open access protocol distributed under the terms of the **Creative Commons Attribution License**, which permits unrestricted use, distribution, and reproduction in any medium, provided the original author and source are credited

Protocol status: Working
We use this protocol and it's working

Created: December 06, 2023

Last Modified: December 05, 2024

Protocol Integer ID: 91874

Keywords: Flow hydrographs, Time series, Hydrograph metrics, Python, Hydrology, Flood

Funders Acknowledgements:
Australian Research Council Linkage grant
Grant ID: LP190100314
Landcare Australia and Hunter-Central Rivers Local Land Services
Australian Commonwealth Government International Research Training Program (iRTP) scholarship
Macquarie University Higher Degree Research Funds

Abstract

The shape characteristics of flow hydrographs hold essential information for understanding, monitoring and assessing changes in flow and flood hydrology at reach and catchment scales. However, the analysis of individual hydrographs is time consuming, making the analysis of hundreds or thousands of them unachievable. A method or protocol is needed to ensure that the datasets being generated, and the metrics produced, have been consistently derived and validated. In this study protocol, we present workflows in Python for extracting flow hydrographs with any available temporal resolution from any Open Access or publicly available gauging station records. The workflow identifies morphologically-defined flow and flood types (i.e. in-channel fresh, high flow and overbank flood) and uses them to classify hydrographs. It then calculates several at-a-station and upstream-to-downstream hydrograph shape metrics including kurtosis, skewness, peak hydrograph stage, peak arrival time, rate-of-rise, peak-to-peak travel time, flood wave celerity, flood peak attenuation, and flood wave attenuation index. Some metrics require GIS-derived data, such as catchment area and upstream-to-downstream channel distance between gauges. The output dataset provides quantified hydrograph shape metrics which can be used to track changes in flow and flood hydrographs over time, or to characterise the flow and flood hydrology of catchments and regions. The workflows are flexible enough to allow for additional hydrograph shape indicators to be added or swapped out, or to use a different hydrograph classification method that suits local conditions. The protocol could be considered a change detection tool to identify where changes in hydrology are occurring and where to target more sophisticated modelling exercises to explain the changes detected. We demonstrate the workflow using 117 Open Access gauging station records that are available for coastal rivers of New South Wales (NSW), Australia.

Guidelines

Please note that this code employs user-defined threshold-based peak identification for distinct flow types and then filters these peaks to ensure they match specific flow criteria. It organises and separates the extracted hydrographs into different folders for each flow type, preventing any mix-ups. Each hydrograph is uniquely processed, named, and saved separately to avoid duplication. Additionally, the code establishes time boundaries around each peak, ensuring that hydrographs are extracted within a defined duration, which helps in accurate assignment and avoids incorrect overlaps or misplacements among different flow types.

The analysis was conducted using Python, leveraging a suite of libraries to efficiently handle data, conduct visualisations, and perform statistical analyses. The analysis was in Python version 3.7 and the script name was 'Hydrograph extraction code - User friendly version.' *Pandas*, a versatile Python library, effortlessly handled streamflow data from CSV and Excel files. It simplified data import, transformation, and management, enabling smooth processing and analysis of the streamflow datasets. The input Excel (.xlsx) file is formatted with two columns: 'date' and 'value.' The 'date' column represents the timestamp of the streamflow measurements, while the 'value' column contains the corresponding streamflow values.

Matplotlib and *Seaborn* were employed for visual representation, generating graphical plots that illustrate the streamflow data over time alongside threshold lines indicating specific flow types (in-channel fresh, high flow, and overbank flood). The code used *datetime* and *timedelta* functionalities to handle the average flood hydrograph duration, allowing adjustments to durations and helping calculate durations for peak segmentation.

The core analysis of the hydrograph peaks was carried out utilising the 'find_peaks' function from *Scipy'sSignal* module. This function enabled the identification of significant peaks within the streamflow data based on user-defined thresholds. Flow hydrograph peaks were categorised into three types: in-channel freshes, high flows, and overbank floods, based on predefined threshold values provided by the user.

The subsequent steps involved the comprehensive visualisation of these identified peaks. This included generating line plots using *Matplotlib* and *Seaborn*, depicting the temporal variation of streamflow. Threshold lines corresponding to different types of flow hydrograph peaks were overlaid on these plots, providing a visual reference for overbank flood, high flow, and in-channel fresh hydrograph peaks within the streamflow data.

Following the visual representation, the analysis delved into the detailed processing of each peak. Around each identified peak, the code segmented the data to capture the dynamics before and after the peak. Specifically, a window of a specified duration (typically 5 days before and after the peak) was defined to confine the hydrograph for further analysis. Within these segments, the code performed some statistical computations, deriving key hydrograph shape metrics such as count, mean, standard deviation, minimum, maximum, kurtosis, and skewness using *Pandas* and *Scipy's* statistical functions. These metrics provided a comprehensive understanding of the characteristics of each identified peak.

In conjunction with the statistical analysis, the code also managed the storage and organisation of extracted hydrographs. It systematically created folders to categorise and store the processed hydrographs and plots these peaks based on their respective flow hydrograph types ('Overbank Flood Peaks', 'High Flow Peaks', 'In-Channel Fresh Peaks') within the time series data, ensuring clear and organised data management throughout the analysis.

Moreover, the code aggregated the statistical outcomes and peak data, combining them into detailed Excel files with .csv extension and image formats for each identified peak ('In_channel_fresh_peaks_stats.csv',' High_flow_peaks_stats.csv', and 'Overbank_flood_peaks_stats.csv'). These comprehensive reports included statistical summaries and plotted representations, contributing to a holistic understanding of the identified peaks.

The required Python packages for extracting hydrograph and assigning them to one of the three flow types (i.e. in-channel fresh, high flow or overbank flood):
- *Pandas*: For managing data using DataFrame structures, enabling CSV and Excel ingestion.
- *Matplotlib*, *Seaborn*: Facilitating data visualisation to represent streamflow and threshold lines graphically.
- *Datetime*, *Timedelta*: Managing temporal data for date modifications and duration calculations.
- *Scipy Signal*: Using `find_peaks` to identify peaks within streamflow data.
- *OS*, *Shutil*: Supporting directory creation and file management.
- *Numpy*: Essential for numerical operations, especially array manipulations and statistical computations.

Materials

1. Python 3.7 (or earlier version) software. This protocol uses Python version 3.7 in Jupyter Notebook platform. Python software is available from:
https://www.python.org/downloads/
Jupyter Notebook is available from:
https://jupyter.org/install
2. ArcGIS Pro. This protocol uses ArcGIS Pro version 3.1. ArcGIS Pro software is available from:
https://pro.arcgis.com/en/pro-app/latest/get-started/download-arcgis-pro.htm
2. Bureau of Meteorology (BoM). Water Data Online. Available from: http://www.bom.gov.au/waterdata/

3. WaterNSW. Continuous Water Monitoring Network. Available from: **https://realtimedata.waternsw.com.au/**
4. NSW Open Access River Styles database. Available from: https://datasets.seed.nsw.gov.au/dataset/new-south-wales-river-styles-spatial-dataset

Before start

1- Users must ensure their input Excel file contains columns labelled 'date' and 'value' for timestamp and streamflow data, respectively, to use this code effectively. Adjustments might be necessary based on specific dataset characteristics and analysis requirements.
2- They need to make sure that Python 3.7 is installed on their systems. The instructions of installing required libraries within Python is provided in Step 6 of this protocol.
3- Always validate the outputs (extracted hydrographs and calculated metrics) and modifications to suit your analysis needs and data format.

A. Overview of steps and flowchart

1 The workflow to quantify flow hydrograph metrics both at-a-station and from upstream-to-downstream
is shown in Fig. 1. This workflow contains 10 main steps:
1) Prepare input data including the hydrographs and geospatial data in Excel.
2) Process gauging station cross-sections to identify the in-channel fresh, high flow and overbank flood stages at each of the gauges in Excel.
3) Input the streamflow time series hydrographs from gauge records into Excel (in NSW these are sourced from the BoM and WaterNSW.
4) Remove redundant data in the excel file of streamflow timeseries.
5) Specify the start and end time of the flow or flood event using the hydrographs in Excel.
6) Run the Python script to extract in-channel fresh, high flow and overbank flood hydrographs.
7) Verify the hydrograph extraction process by visual observation and adjust the inputs (remove outliers and adjust start time and end time) if needed in the Python script.
8) Calculate hydrograph metrics; kurtosis (Eqn 1), skewness (Eqn 2), peak flow stage, peak flow date and rate-of-rise (RoR) (Eqn 3) for each hydrograph in Python.
9) Calculate contributing catchment area (A_c), and upstream-to-downstream channel distance between gauges (D) in ArcGIS Pro and input them into the Python script (in NSW these metrics are calculated by using NSW River Styles streamlines layer (44) in ArcGIS Pro (36)).
10) Calculate peak-to-peak travel time (t_T) (Eqn 4), flood wave celerity (C) (Eqn 5), flood peak attenuation (H_a) (Eqn 6) and flood wave attenuation index (FWAI) (Eqn 7) in Excel.
Please note that Steps 8 and 10 produce a hydrograph metrics database as an output (See Fig. 2).

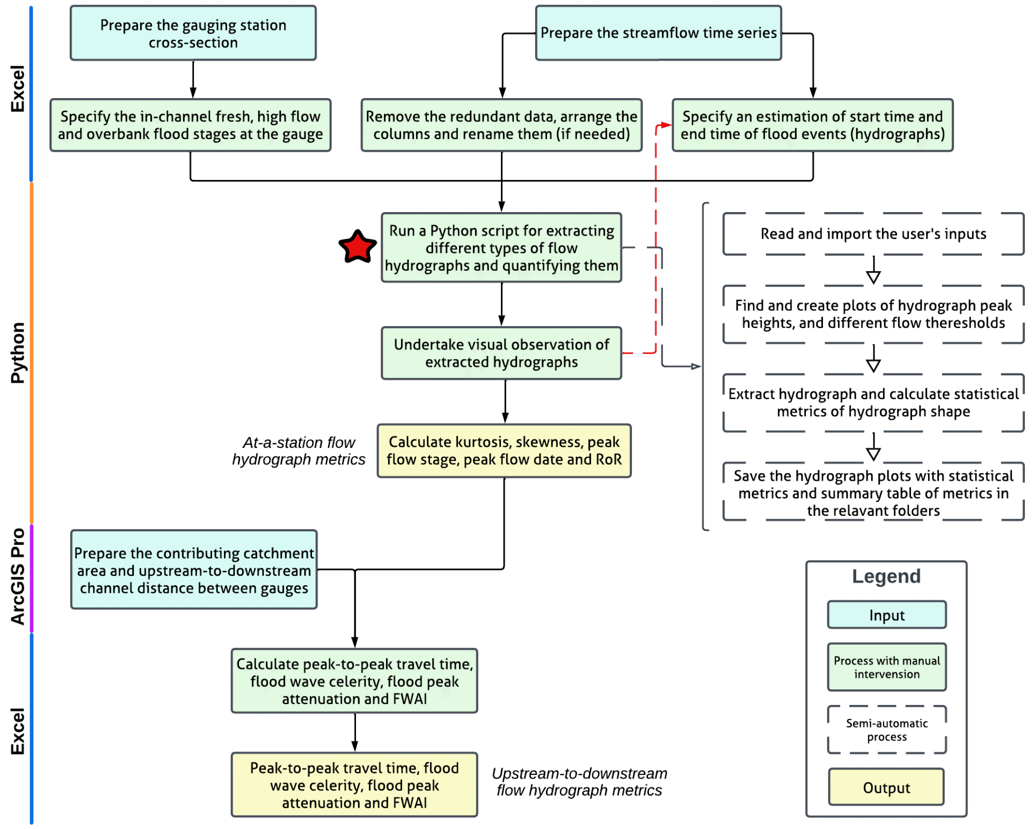

Fig. 1 Flowchart showing the full methodology for extracting and quantifying hydrographs from the streamflow time series at each gauge. The yellow boxes produce a metrics database as an output. The Python script indicated by a red star, is detailed in the protocols.io that accompanies this paper. Data source: BoM, WaterNSW, ESRI and NSW River Styles. Green boxes and white boxes denote parts of the protocol that require manual intervention by a user and semi-automatic parts, respectively.

B. Input data preparation

2 **Hierarchical structure of the hydrograph analysis protocol**

Fig. 2 shows the hierarchical structure of the hydrograph analysis protocol presented in this study. It involves structured-based preparing, calculating, organising and saving data that captures different information about hydrological characteristics. This structure aims to provide a comprehensive and organised representation of metrics derived from hydrographs and geospatial data, allowing for detailed analysis and interpretation.

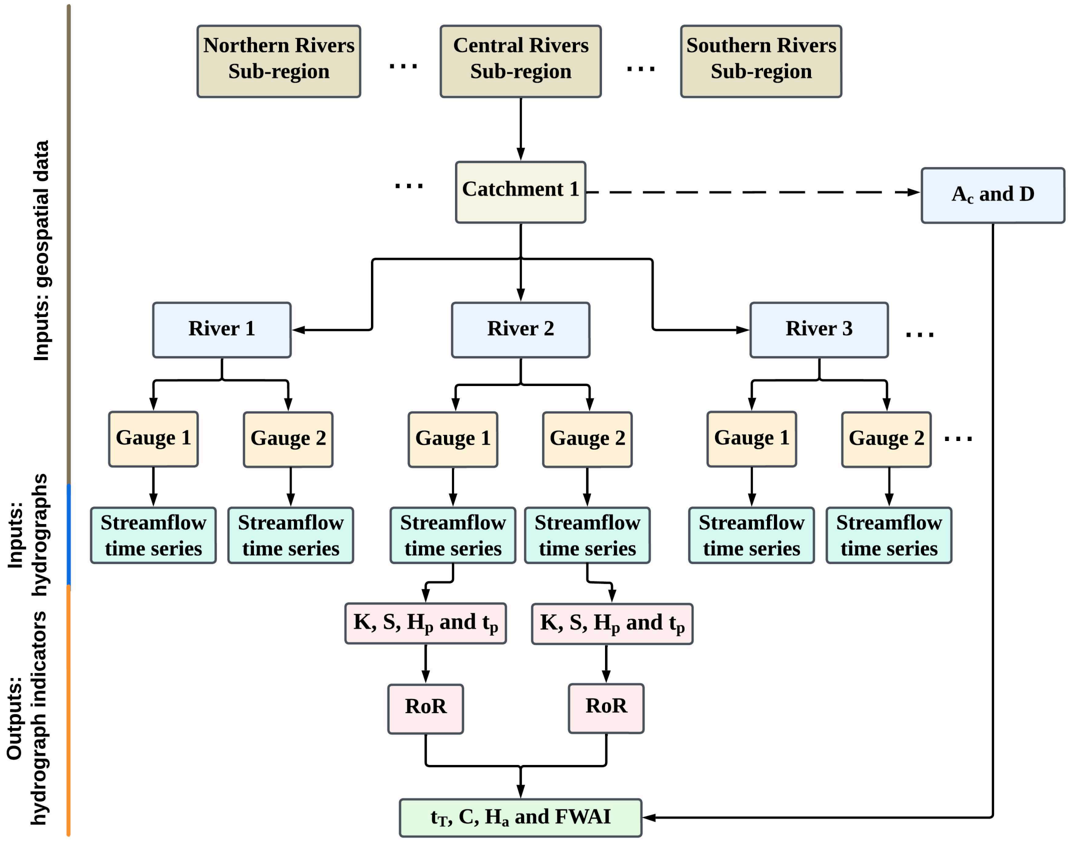

Fig 2. Hierarchical structure of the hydrograph analysis protocol. Data source: BoM, WaterNSW, ESRI and NSW River Styles.

3 **Download the streamflow time series from BoM and WaterNSW.**

The streamflow data at sub-1hour intervals is available from the Australian Bureau of Meteorology (BoM) (Fig 3) and WaterNSW 2023 (Fig 4) Open Access portals.

WaterNSW Website

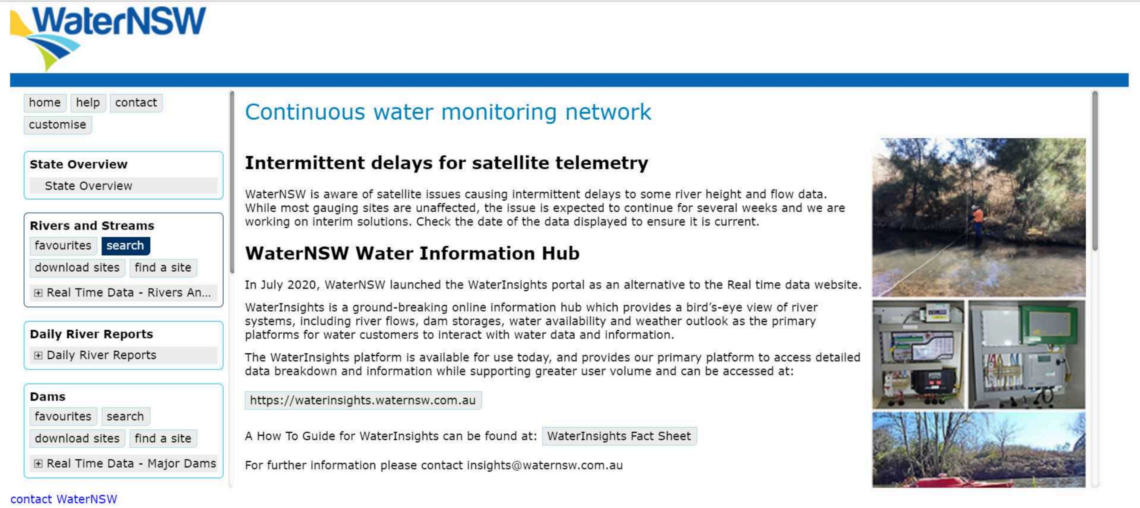

Fig 3. WaterNSW Website (6 December 2023).

BoM Website

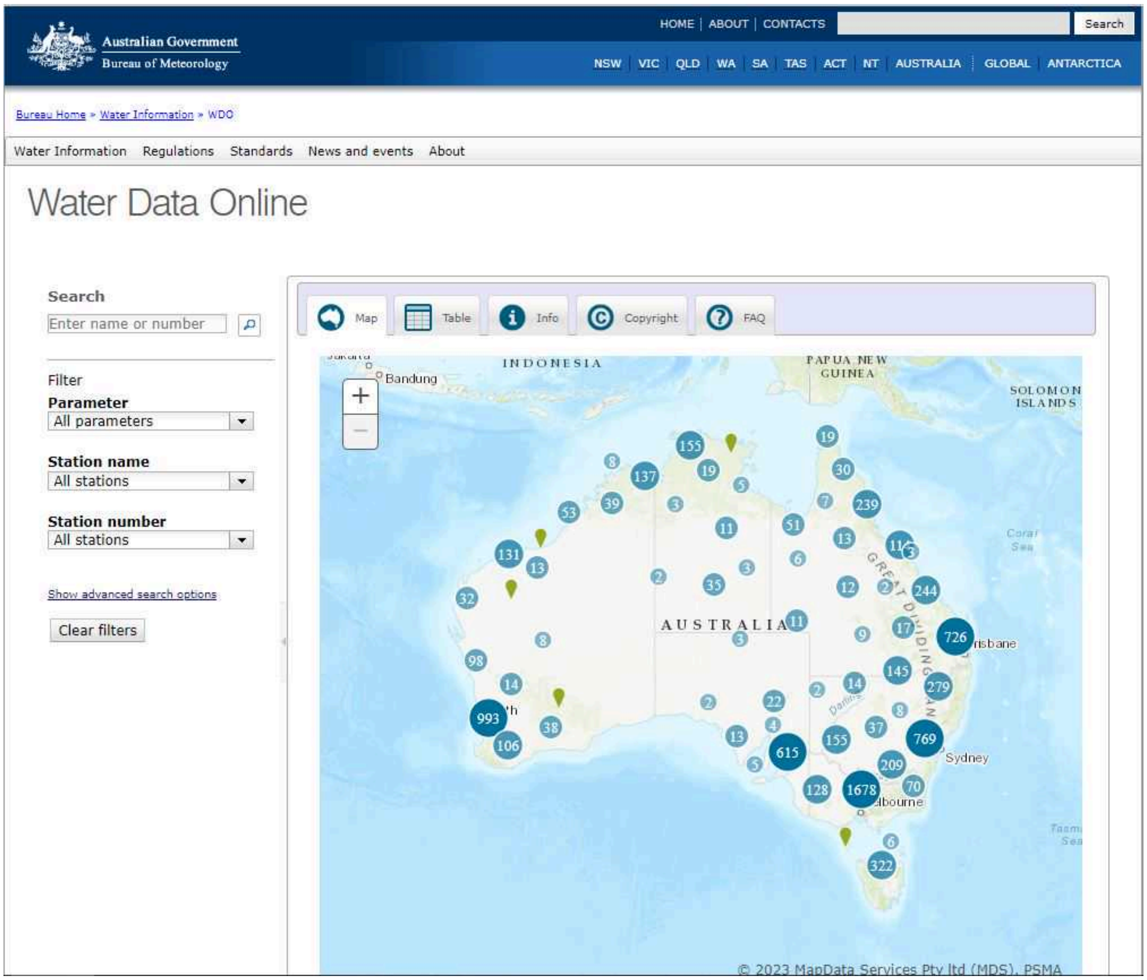

Fig 4. BoM Website (6 December 2023).

4 **Remove the redundant data in the excel file of streamflow timeseries.**

In different places and for different gauges, stage height data may come to a user in different formats. Therefore, setting up the input data excel spreadsheet to a standard format is necessary. For example, the streamflow data download from BoM and WaterNSW portals for NSW streams needs to be adjusted and filtered before importing into the Python code. The label of the first column and row (cell A1) should be 'date' and the second column first row (B1) should be 'value'. All other redundant information should be removed from the excel file. Then, this file should be saved in .xlsx format. The date data interval is 1 hour and the unit of stage height is meters. The format of the date is dd/mm/yyyy hh:mm:ss.

date	value
1/01/1913 13:00	0.305
1/01/1913 14:00	0.305
1/01/1913 15:00	0.305
1/01/1913 16:00	0.305
1/01/1913 17:00	0.305
1/01/1913 18:00	0.305
1/01/1913 19:00	0.305
1/01/1913 20:00	0.305
1/01/1913 21:00	0.305
1/01/1913 22:00	0.305
1/01/1913 23:00	0.305
2/01/1913 0:00	0.305
2/01/1913 1:00	0.305
2/01/1913 2:00	0.305
2/01/1913 3:00	0.305

Fig 5. The input streamflow data example

5 **Specify the in-channel fresh, high flow and overbank flood stages at the study gauges.**

Hydrologists and river experts often classify flow stage heights into categories based on water level at each cross-section. This protocol runs on stage height hydrographs instead of discharge for several reasons including, greater data availability, ease of comparison across different streams, ease of visualisation, and to match to stakeholders' experience in the field and emergency warnings that are issued during floods. Also, each river has a different morphology and therefore discharge or recurrence intervals for say a bankfull flow are not consistent across the landscape. Therefore, this protocol runs on three morphologically-defined flow and flood stages which can then be used to undertake like-with-like comparisons of flow and flood behaviour across catchments and regions. The three flow stages are: in-channel fresh, high flow and overbank flood. Table 1 shows definitions of different flow stages, and Fig 6 represents different flow thresholds at a conceptual cross-section.

Flow stage class	Definition of the flow stage
Overbank flood	A stage height that is 5% above the bankfull flow absolute threshold.

Flow stage class	Definition of the flow stage
In-channel fresh	A stage height that is 95% higher than the low flow absolute threshol d (7Q10) and 50% higher than the fresh flow absolute threshold (half bankfull stage height).
High flow	A stage height that is 50% lower than the bankfull flow absolute thre shold (top of the macrochannel bank) and 5% higher than this thresh old.

Table 1. definitions of different flow stages (Arash et al., 2023)

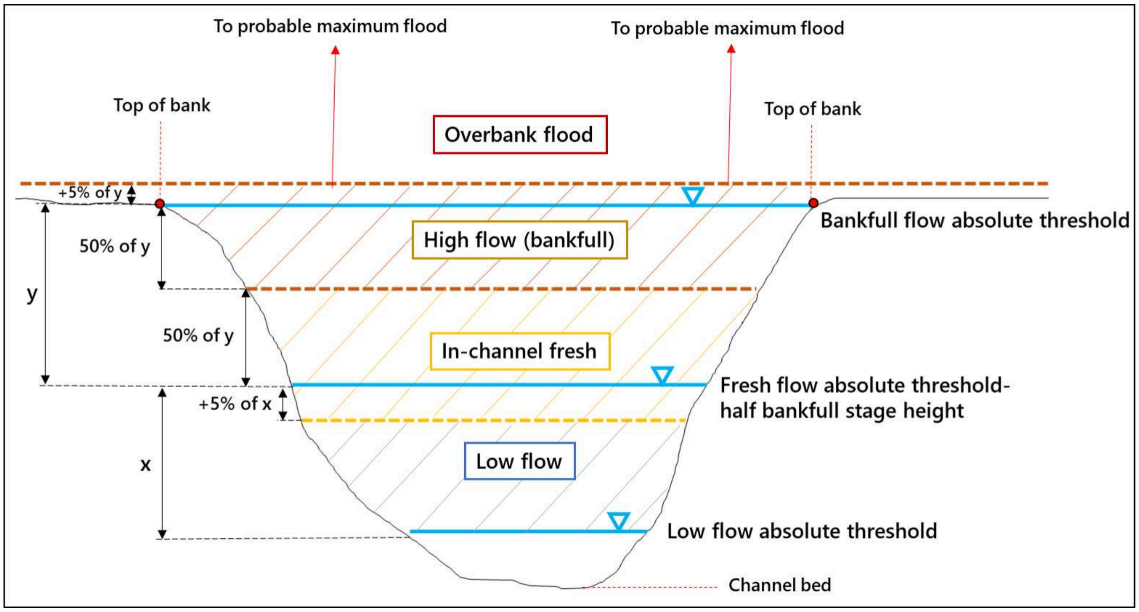

Fig 6. Schematic cross-section showing stage height thresholds and flow types used in this paper (Arash et al., 2023).

6 **Determine the start and end time of flood hydrograph.**

Preparation of other inputs include identification of the in-channel fresh, high flow and overbank flood stages at each gauge station and identification of the start time and end time of each flow or flood event of interest. The start time would typically be the onset of the precipitation event, which causes a rise in stage height. The end time normally indicates a return to base flow level. To identify the start and end times, flow record variability, local flow conditions and the purpose of study should be considered. In this study, 10 days was selected as the duration of flow and flood events. For ease, this is defined as 5 days back from the flow peak and 5 days forward from the flood peak (Fig 7).

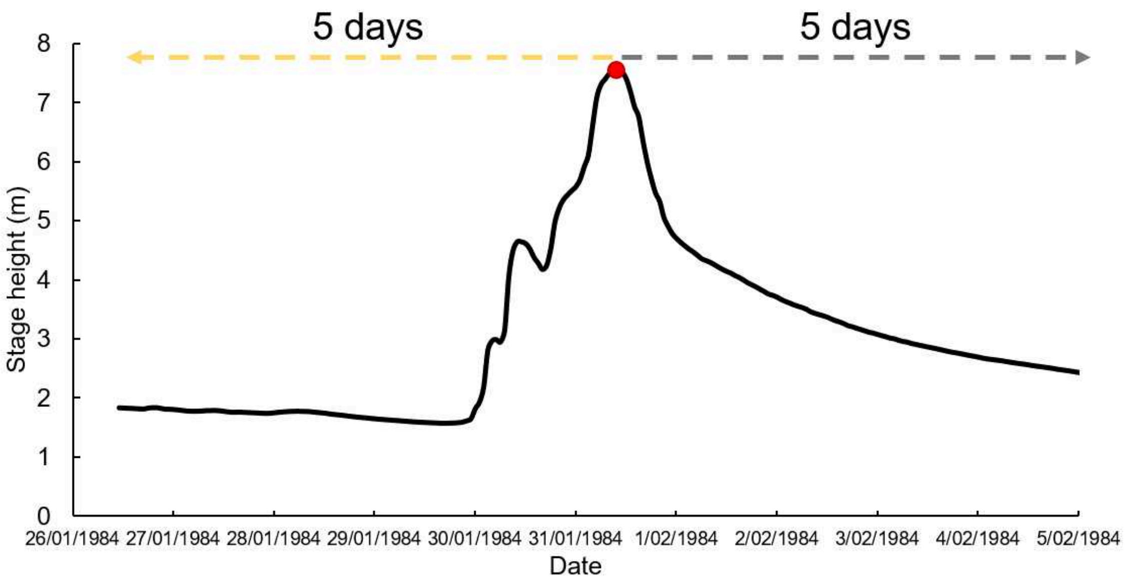

Fig 7. Illustration of the selection of duration of flow hydrographs.

C. Extraction and quantification of flow hydrographs from streamflow time series.

7 **Introduction:**

Extract the in-channel fresh, high flow and overbank flood hydrographs by running a Python script, and calculate flow indicators (kurtosis, skewness and peak flow stage) for each flow hydrograph.

By running the Python code, the script extracts flow hydrographs and calculates kurtosis, skewness, peak value, peak date and some other statistics for each hydrograph. Before undertaking further analysis, the extracted hydrographs need to be checked to ensure that the delineated hydrographs and calculated metrics are correct and any inputs (streamflow time series, flow stages, and duration of hydrographs) adjusted where needed.

In Excel, RoR is calculated using peak value and peak date in Eqn 3. Total drainage area draining into each gauge and total area of each catchment is calculated using the watershed tool
in ArcHyro extension in ArcGIS Pro (Arash et al., 2023). Subcatchment area draining to each gauge is classified as small (e.g. <2000 km^2), moderate (e.g. 2,000-10,000 km^2) and large (e.g. >10,000 km^2). Also, gauges are classified as upstream (a gauge captured 10% of the total catchment area), midstream (11%–50%), and downstream (>51%).

Peak-to-peak travel time of each pair of hydrographs is calculated on the peak value at the upstream and downstream gauges. The distance between upstream and downstream gauge pairs
is calculated in ArcGIS Pro by using any readily available, accurate, streamlines layer (in NSW this is the River Styles streamlines layer). The distance between gauge pairs and peak-

to-peak travel time are used to calculate flood wave celerity. FWAI is calculated using the peak stage height value and contributing drainage area at the upstream and downstream gauges.

8 **Pre-processing step:**

This is a script that checks for required Python libraries, prompts the user for installation if any are missing, and handles the installation process based on the user's choice.

```
import subprocess

libraries = [
    "pandas",
    "matplotlib",
    "seaborn",
    "scipy",
    "numpy"
]

missing_libraries = []

for lib in libraries:
    try:
        __import__(lib)
    except ImportError:
        missing_libraries.append(lib)

if missing_libraries:
    print("The following libraries are missing: ")
    for lib in missing_libraries:
        print(lib)

    install_choice = input("Would you like to install the missing
libraries? (y/n): ")

    if install_choice.lower() == 'y':
        for lib in missing_libraries:
            subprocess.call(['pip', 'install', lib])
        print("Libraries installed successfully!")
    else:
        print("Libraries were not installed.")
else:
    print("All required libraries are installed.")
```

9 **The main processes:**

The initial code segments primarily focused on data manipulation, threshold-based peak detection using `find_peaks`, and visual representation using Seaborn and Matplotlib libraries. These segments included functions to manage user input for thresholds, data loading, and graphical visualization, offering a comprehensive approach to visualize and identify specific types of peaks in a time series dataset.

Subsequently, the code delved deeper into peak processing, statistical analysis, and data saving. It included a function to process and save peak values, generate statistics, create Excel files and plots for each peak instance, as well as combining and storing summary statistics in separate CSV files for further analysis. This section extended the analysis to encompass detailed statistical insights and organized data into structured formats for comprehensive analysis and reporting.

9.1 **Essential Python Libraries for Data Analysis and Visualization:**

These following lines of code are used to import specific Python libraries/modules that serve various purposes (data analysis, visualization, statistical computations, file operations, and mathematical calculations):

```
import pandas as pd
import matplotlib.pyplot as plt
from datetime import datetime, timedelta
import seaborn as sns
from scipy.signal import find_peaks
import os
import shutil
from scipy.stats import kurtosis, skew
import numpy as np
```

9.2 **Threshold Input and Data Loading Functions for Flood Analysis:**

The following snippet is a combination of functions for user input retrieval (`get_user_input()`) and data loading (`pd.read_excel`) to work with streamflow data stored in CSV or Excel format, using Pandas DataFrames for data manipulation and analysis.

```
# Function to get user input for thresholds and duration.
def get_user_input():
    overbank_threshold = float(input("Enter the Overbank flood
threshold (m): "))
    high_flow_threshold = float(input("Enter the High flow
threshold (m): "))
    in_channel_threshold = float(input("Enter the In-channel fresh
threshold (m): "))
    time_delta = int(input("Enter the average flood hydrograph
duration (in days): "))
    return overbank_threshold, high_flow_threshold,
in_channel_threshold, time_delta

# Converts streamflow time series in csv format to Pandas
dataframe.
def csv_to_dataframe(data_file):
    time_data = pd.read_csv(data_file)
    return time_data

# Function to load data from an excel file.
data_file = '210001.xlsx'
time_data = pd.read_excel(data_file, names=['date', 'value'])

# Get user input for thresholds and timedelta.
overbank_threshold, high_flow_threshold, in_channel_threshold,
time_delta = get_user_input()
```

9.3 **Peak Detection for Flood Analysis and Time Modification Functions:**

The code segment demonstrates the use of the `find_peaks` function from `scipy.signal` to identify peaks in a time series dataset ('values'). It employs predefined thresholds to isolate specific types of peaks related to flood levels. It also contains a function, `modify_time`, that adjusts dates in the dataset based on certain conditions, ensuring they fall within a specified range.

```
# Example of finding peaks using find_peaks from scipy.signal
based on thresholds (assuming 'value' column holds the data).
values = time_data['value']
prominence = 10
Overbank_flood_peaks, _ = find_peaks(values,
height=overbank_threshold, distance=10)
High_flow_peaks, _ = find_peaks(values,
height=high_flow_threshold, distance=10)
In_channel_fresh_peaks, _ = find_peaks(values,
height=in_channel_threshold, distance=10)

In_channel_fresh_peaks = np.intersect1d(In_channel_fresh_peaks,
np.where((values >= in_channel_threshold) & (values <=
high_flow_threshold)))
High_flow_peaks = np.intersect1d(High_flow_peaks, np.where((values
>= high_flow_threshold) & (values <= overbank_threshold)))
Overbank_flood_peaks = np.intersect1d(Overbank_flood_peaks,
np.where(values >= overbank_threshold))

# Function to modify time.
def modify_time(date_time):
    if date_time.year > datetime.now().year:
        date_time = date_time - timedelta(days=36525)
    return date_time

# Modify time column in dataframe (apply modify_time function to
'date' column in time_data).
time_data['date'] = pd.to_datetime(time_data['date'])
time_data['date'] = time_data['date'].apply(lambda x:
modify_time(x))

def modify_time(date_time):
    if date_time.year>datetime.now().year:
        date_time=date_time-timedelta(days=36525)
    return date_time
time_data['date']=time_data['date'].apply(lambda x :
modify_time(x))# change object time to date time time stamp
time_data['date'].iloc[0]
modify_time(time_data['date'].iloc[-1])
```

9.4 **Visualizing Hydrograph Peaks and Thresholds withSeaborn and Matplotlib:**

This code segment illustrates graph plotting using Seaborn and Matplotlib libraries. It visualizes a time series dataset (`time_data`) representing stage heights, along with

threshold lines based on user-defined input. Additionally, it organizes extracted hydrographs into separate folders based on peak types (overbank flood, high flow, and in-channel fresh).

```
# Plotting the graph using Seaborn and Matplotlib.
a4_dims = (11.7, 8.27)
fig, ax = plt.subplots(figsize=a4_dims)
sns.lineplot(data=time_data, x='date', y='value')

# Adding threshold lines and labels based on user input (adds
threshold lines to the plot).
plt.axhline(y=overbank_threshold, color='r', linestyle='--',
label=f'Overbank Flood Threshold: {overbank_threshold}')
plt.axhline(y=high_flow_threshold, color='b', linestyle='-.',
label=f'High Flow Threshold: {high_flow_threshold}')
plt.axhline(y=in_channel_threshold, color='g', linestyle=':',
label=f'In-channel Fresh Threshold: {in_channel_threshold}')

# Displaying the legend with all the labels
plt.legend()

# Creates folders if these folders don't exist or delete it if
there were folders before.
def create_folders(peak_path):
    if os.path.exists(peak_path):
        shutil.rmtree(peak_path)
    os.mkdir(peak_path)

# Creates three folders to put the extracted hydrographs in them
(for different types of peaks).
folders = ["Overbank_flood_peaks", "High_flow_peaks",
"In_channel_fresh_peaks"]
for folder in folders:
    create_folders(folder)

# Indicate in-channel fresh peaks, high flow peaks, and overbank
flood peaks in time series.
fig, axs = plt.subplots(3, 1, figsize=(11.7, 8.27))

for i, peaks in enumerate([Overbank_flood_peaks, High_flow_peaks,
In_channel_fresh_peaks]):
    sns.scatterplot(x=time_data['date'].iloc[peaks],
y=time_data['value'].iloc[peaks], palette="deep", ax=axs[i])
    sns.lineplot(data=time_data, x='date', y='value',
palette="deep" if i != 2 else "rocket", ax=axs[i])
    axs[i].grid(True)
    axs[i].set_xlabel('Date')
    axs[i].set_ylabel('Stage Height (m)')
```

```
    titles = ['Overbank Flood Peaks', 'High Flow Peaks', 'In-
Channel Fresh Peaks']
    axs[i].set_title(titles[i])

plt.tight_layout()
plt.show()
```

9.5 **Peak Value Processing, Statistics Generation, and File Saving:**

This code section defines a function `process_peak_values()` responsible for processing, analyzing, and saving peak values obtained from different types of flood peaks identified earlier (`Overbank_flood_peaks`, `High_flow_peaks`, `In_channel_fresh_peaks`). It performs statistics generation, Excel file creation, and image plotting for each peak instance based on a specified time window (`time_delta`). Additionally, it combines and saves summary statistics for each peak type into separate CSV files.

```
# Process and save peak values (function to process peak values,
generate statistics, save Excel files, and plots).
def process_peak_values(peaks, folder_name, time_data):
    for idx, peak_value in
enumerate(time_data['date'].iloc[peaks]):
        lower_bound = pd.to_datetime(peak_value) -
timedelta(days=time_delta/2)
        upper_bound = pd.to_datetime(peak_value) +
timedelta(days=time_delta/2)
        mask = (time_data['date'] > lower_bound) &
(time_data['date'] < upper_bound)
        a4_dims = (11.7, 8.27)
        fig, ax = plt.subplots(figsize=a4_dims)

        d = {'date': time_data[mask]['date'], 'value':
time_data[mask]['value']}
        df_peaks = pd.DataFrame(data=d)
        df_peaks['stat'] = "*****"
        df_peaks['stat_vaue'] = "*****"
        stats = pd.DataFrame(df_peaks.describe())
        stats.reset_index(inplace=True)

        additional_stats = [{'index': 'kurtosis', 'value':
kurtosis(np.array(df_peaks['value']))},
            {'index': 'skewness', 'value':
skew(np.array(df_peaks['value']))}]
        additional_stats_df = pd.DataFrame(additional_stats)
        stats = pd.concat([stats, additional_stats_df],
ignore_index=True)

        stats_to_find = ['count', 'mean', 'std', 'min', '25%',
'50%', '75%', 'max', 'kurtosis', 'skewness']
        for i in range(len(stats.index)):
            df_peaks.iloc[i, 2] = stats_to_find[i]
            df_peaks.iloc[i, 3] = stats.iloc[i, 1]

        df_peaks.to_excel(f'{folder_name}/flood_{str(idx)}.xlsx')
        plot = sns.lineplot(time_data[mask]['date'],
time_data[mask]['value'])
        fig = ax.get_figure()
        fig.savefig(f'{folder_name}/flood_{str(idx)}.jpg')

# Plotting and saving peak data.
```

```
process_peak_values(Overbank_flood_peaks, 'Overbank_flood_peaks',
time_data)
process_peak_values(High_flow_peaks, 'High_flow_peaks', time_data)
process_peak_values(In_channel_fresh_peaks,
'In_channel_fresh_peaks', time_data)
plt.show()

# Combining and saving summary statistics from processed peak
data.
folders = ['Overbank_flood_peaks', 'High_flow_peaks',
'In_channel_fresh_peaks']
columns = ['count', 'mean', 'std', 'min', '25%', '50%', '75%',
'max', 'kurtosis', 'skewness']
root_path = os.getcwd()

for folder in folders:
    path = os.path.join(root_path, folder)
    text_files = [os.path.join(path, f) for f in os.listdir(path)
if f.endswith('.xlsx')]
    summary_data = []

    for file in text_files:
        df_temp = pd.read_excel(file)
        file_stats = [df_temp.iloc[i]['stat_vaue'] for i in
range(len(columns))]
        half_time_delta = int(time_delta / 2)
        midpoint_date = df_temp.iloc[-1]['date'] -
timedelta(days=half_time_delta)
        file_stats.extend([df_temp.iloc[0]['date'], midpoint_date,
df_temp.iloc[-1]['date']])
        summary_data.append(file_stats)

    stats_df = pd.DataFrame(summary_data, columns=columns +
['start_date', 'peak_date', 'end_date'])
    stats_df.to_csv(f"{folder}_stats.csv", index=False)
```

D. Expected results and technical verification

10 **Expected results:**

This script creates folders and generates separate subplots for different types of identified hydrographs (Overbank Flood Peaks, High Flow Peaks, In-Channel Fresh Peaks) within the time series. These subplots showcase the peaks overlaid on the original data for visual examination.

Besides, it calculates important statistics (kurtosis, skewness, peak value, peak date and some other statistics) for each hydrograph that can be used for further analysis and reference (Table 2). Fig 8 shows an example of the application of script to extract and quantify flow hydrographs for the in-channel fresh, high flow and overbank flood flow stage classes from streamflow time series data for the Hunter River at Singleton gauging station from 01/01/1913 to 21/11/2023.

Gauge	Flood Type	Gauge-Flood Type	Number	name	max	kurtosis	skewness	peak_date	Contributing Drainage Area (Sq.Km)
210004	High flow	210004-High flow	0	flood_0.xlsx	5.92	-0.71	0.72	25/02/1908 6:00	1672
210004	In-channel fresh	210004-In-channel fresh	0	flood_0.xlsx	4.25	-0.60	0.74	16/03/1908 7:00	1672
210004	High flow	210004-High flow	1	flood_1.xlsx	5.47	-1.54	-0.12	16/01/1910 7:00	1672
210006	Overbank flood	210006-Overbank flood	0	flood_0.xlsx	4.50	2.64	1.88	15/05/1913 9:00	3340
210001	Overbank flood	210001-Overbank flood	0	flood_0.xlsx	14.19	-0.37	1.03	16/05/1913 10:00	16400
210004	In-channel fresh	210004-In-channel fresh	1	flood_1.xlsx	3.35	-1.35	0.05	12/06/1913 8:00	1672
210004	High flow	210004-High flow	11	flood_2.xlsx	5.31	0.45	1.31	1/07/1913 8:00	1672
210001	In-channel fresh	210001-In-channel fresh	0	flood_0.xlsx	7.16	-0.84	0.78	1/07/1913 10:00	16400
210004	In-channel fresh	210004-In-channel fresh	12	flood_2.xlsx	4.26	-0.18	0.82	8/05/1915 11:00	1672
210006	In-channel fresh	210006-In-channel fresh	0	flood_0.xlsx	1.99	1.51	1.65	23/02/1916 9:00	3340
215002	High flow	215002-High flow	0	flood_0.xlsx	7.61	-0.32	1.00	5/10/1916 8:00	1450
210001	In-channel fresh	210001-In-channel fresh	12	flood_2.xlsx	5.99	-0.71	0.77	14/12/1916 10:00	16400
210006	In-channel fresh	210006-In-channel fresh	1	flood_1.xlsx	1.90	3.53	2.13	25/10/1919 9:00	3340
210004	In-channel fresh	210004-In-channel fresh	23	flood_3.xlsx	3.79	0.08	1.28	2/07/1920 8:00	1672
210006	Overbank flood	210006-Overbank flood	1	flood_1.xlsx	6.99	0.25	1.17	2/07/1920 8:00	3340
210006	In-channel fresh	210006-In-channel fresh	0	flood_0.xlsx	6.99	0.25	1.17	2/07/1920 8:00	3340
210001	High flow	210001-High flow	0	flood_0.xlsx	9.98	-1.08	0.15	2/07/1920 11:00	16400
210006	High flow	210006-High flow	0	flood_0.xlsx	3.62	3.34	2.10	10/12/1920 9:00	3340
210001	In-channel fresh	210001-In-channel fresh	23	flood_3.xlsx	6.38	-1.32	0.26	18/12/1920 10:00	16400

Table 2. Calculated hydrograph metrics (i.e., peak value, kurtosis, and skewness)

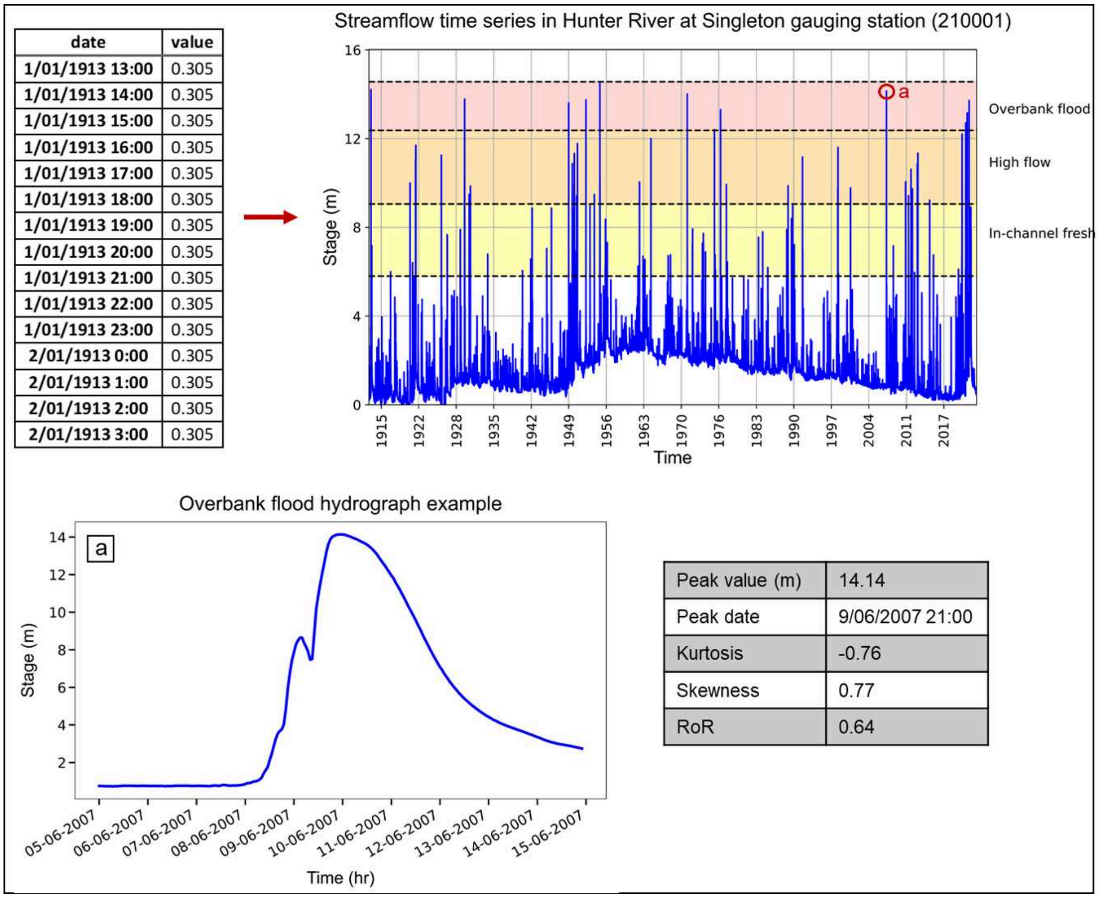

Fig 8. An example of script results for extracting flow hydrographs from the stream flow time series at the Hunter River at Singleton gauging station (the output metrics table is in the database).

11 **Verify the hydrograph extraction process by visual observation and adjust the inputs, if needed.**

The comparative analysis is not available for this study. Nevertheless, the script was executed iteratively, allowing for manual observation of the generated hydrographs. This ensured that the specified inputs consistently produced hydrographs that align with real-world expectations. Additionally, thorough checks were implemented to prevent the duplication of hydrographs, and mixed outputs in the generated dataset (see Fig 9).

Besides, the streamflow time series sourced by BoM and WaterNSW and used for extracting hydrographs are of high quality (McMahon and Peel, 2019). The hydrograph indicators calculated in this study were successfully used in previous studies for decadal time-series changes in flow hydrology as an indicator of flood mitigation signal and natural flood management potential (e.g., Fryirs et al., 2023; Arash et al., 2023).

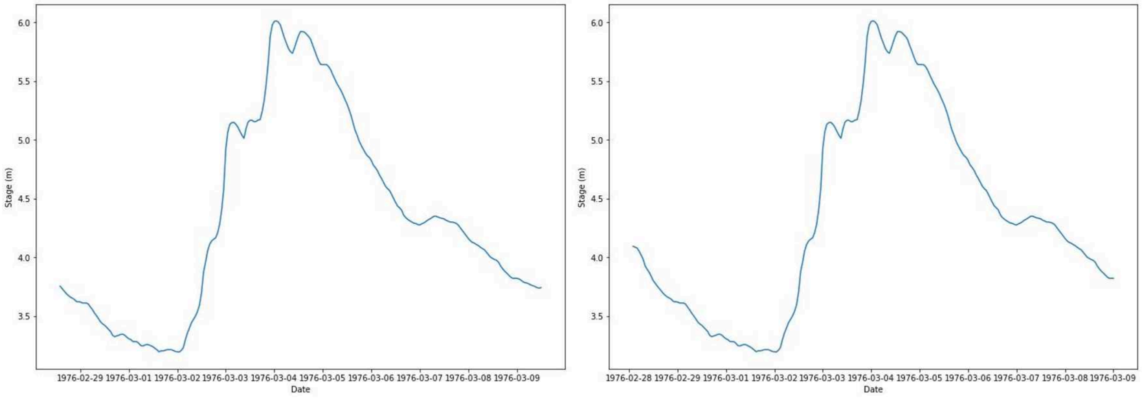

Fig 9. An example of a duplicated hydrograph in first run of the code in the database.

12 **Calculate RoR, t_T, C, H_a and FWAI:**

The calculated statistics in Step 11 were used to calculate rate-of-rise (RoR), peak-to-peak travel time (t_T), flood wave celerity (C), flood peak attenuation (H_a) and flood wave attenuation index (FWAI) by using contributing catchment area (Ac), and upstream-to-downstream channel distance between gauges (D) derived from the NSW River Styles database (2023) in ArcGIS Pro version 3.2 (Figs 10-11; Table 3).

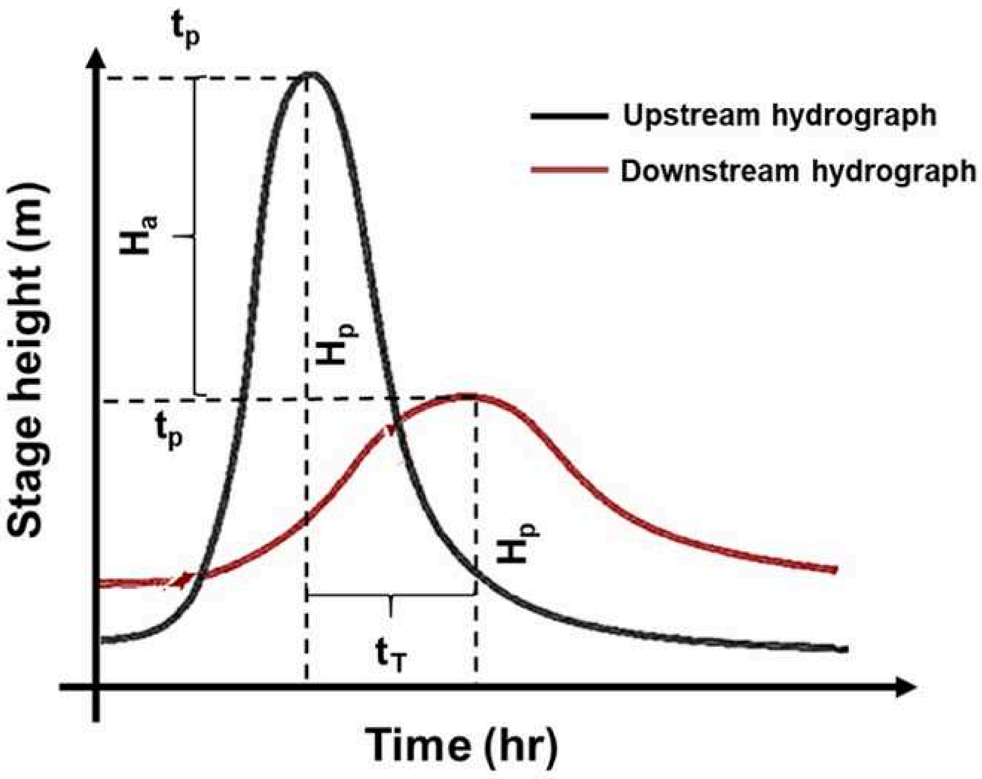

Fig 10. Schematic shape of upstream and downstream hydrographs with hydrograph metrics (H_p: peak hydrograph stage (m), H_a: flood peak attenuation (m); t_T: peak-to-peak travel time (hr); and t_p: peak arrival time (hr)).

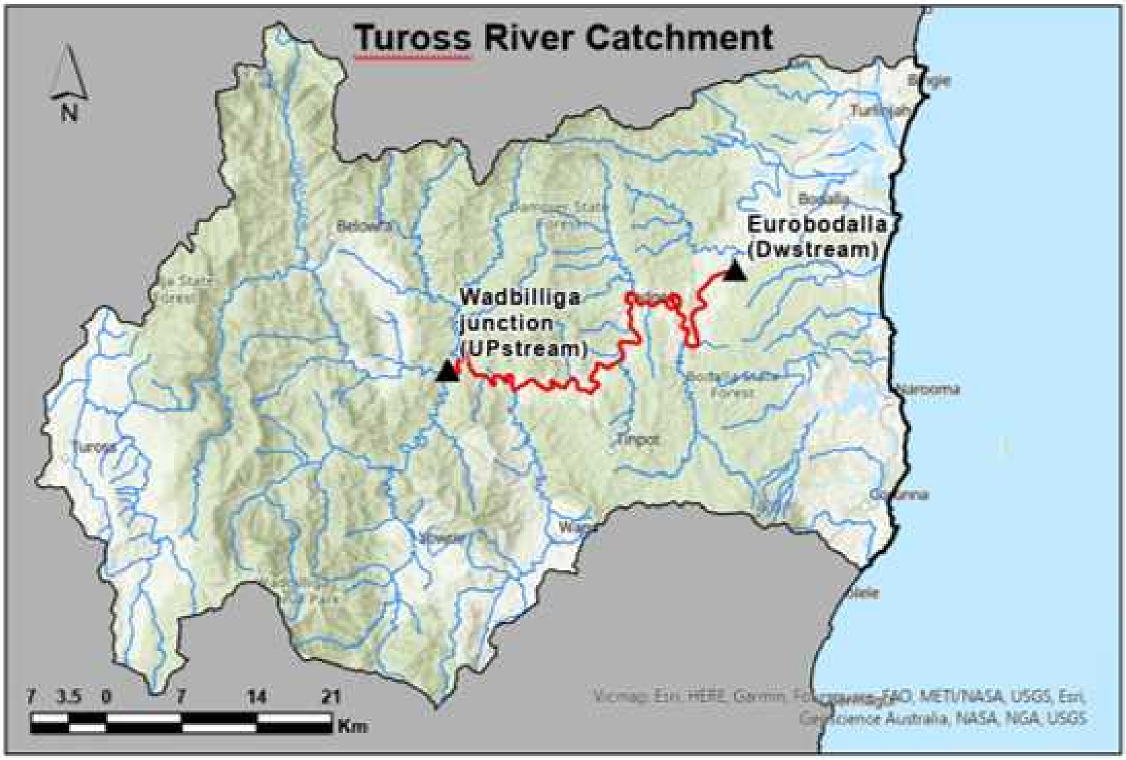

Fig 11. An example of a pair of upstream to downstream gauges along the Tuross River.

Gauge	Flood Type	Gauge-Flood Type	Pair	Travel Time (hr)	Celerity (m/s)	FWAI	Flood Attenuation (m)	Distance(m)	Peak Date (numerical)	RoR
203005	In-channel fresh	203005-In-channel fresh	1	18	1.47	-0.0019	-2.10	95396.96	1972.17	0.40
203004	High flow	203004-High flow	1						1972.17	0.50
203005	In-channel fresh	203005-In-channel fresh	1	30	0.88	-0.0006	-0.69	95396.96	1972.25	0.33
203004	In-channel fresh	203004-In-channel fresh	1						1972.25	0.36
203005	In-channel fresh	203005-In-channel fresh	1	9	2.94	-0.0027	-2.97	95396.96	1972.83	0.37
203004	High flow	203004-High flow	1						1972.83	0.50
203005	High flow	203005-High flow	1	25	1.06	-0.0008	-0.84	95396.96	1974.08	0.55
203004	Overbank flood	203004-Overbank flood	1						1974.08	0.58
203005	In-channel fresh	203005-In-channel fresh	1	26	1.02	-0.0022	-2.40	95396.96	1974.50	0.37
203004	High flow	203004-High flow	1						1974.50	0.48
203005	High flow	203005-High flow	1	12	2.21	-0.0023	-2.51	95396.96	1975.25	0.50
203004	Overbank flood	203004-Overbank flood	1						1975.25	0.61
203005	High flow	203005-High flow	1	15	1.77	-0.0004	-0.45	95396.96	1976.00	0.51
203004	Overbank flood	203004-Overbank flood	1						1976.00	0.53
203005	In-channel fresh	203005-In-channel fresh	1	120	0.22	-0.0001	-0.08	95396.96	1976.08	0.36
203004	In-channel fresh	203004-In-channel fresh	1						1976.08	0.37
203005	Overbank flood	203005-Overbank flood	1	19	1.39	0.0008	0.85	95396.96	1976.17	0.68
203004	Overbank flood	203004-Overbank flood	1						1976.17	0.64

Table 3. Calculated hydrograph metrics (i.e., RoR, t_T, C, H_a and FWAI)

Protocol references

1. Python 3.7 (or earlier version) software. This protocol uses Python version 3.7 in Jupyter Notebook platform. Python software is available from:
https://www.python.org/downloads/

Jupyter Notebook is available from: https://jupyter.org/install

2. ArcGIS Pro. This protocol uses ArcGIS Pro version 3.2. ArcGIS Pro software is available from:
https://pro.arcgis.com/en/pro-app/latest/get-started/download- arcgis-pro.htm

2. Bureau of Meteorology (BoM). Water Data Online. Available from: **http://www.bom.gov.au/waterdata/**

3. WaterNSW. Continuous Water Monitoring Network. Available from: **https://realtimedata.waternsw.com.au/**

4. NSW Open Access River Styles database. Available from: https://datasets.seed.nsw.gov.au/dataset/new-south-wales-river- styles-spatial-dataset
